# A Comprehensive Survey on the Integrity of Localization Systems

**DOI:** 10.3390/s25020358

**Published:** 2025-01-09

**Authors:** Elias Maharmeh, Zayed Alsayed, Fawzi Nashashibi

**Affiliations:** 1 Valeo Mobility Tech Center (VMTC), 6 Rue Daniel Costantini, 94000 Créteil, France; zayed.alsayed@valeo.com; 2 Inria-ASTRA Team, 48 Rue Barrault, 75013 Paris, France; fawzi.nashashibi@inria.fr

**Keywords:** integrity, protection level, localization, SLAM, fault/outlier detection, robust optimization, factor graph, fault detection and exclusion (FDE), model-based FDE, coherence-based FDE

## Abstract

This survey extends and refines the existing definitions of integrity and protection level in localization systems (localization as a broad term, i.e., not limited to GNSS-based localization). In our definition, we study integrity from two aspects: quality and quantity. Unlike existing reviews, this survey examines integrity methods covering various localization techniques and sensors. We classify localization techniques as optimization-based, fusion-based, and SLAM-based. A new classification of integrity methods is introduced, evaluating their applications, effectiveness, and limitations. Comparative tables summarize strengths and gaps across key criteria, such as algorithms, evaluation methods, sensor data, and more. The survey presents a general probabilistic model addressing diverse error types in localization systems. Findings reveal a significant research imbalance: 73.3% of surveyed papers focus on GNSS-based methods, while only 26.7% explore non-GNSS approaches like fusion, optimization, or SLAM, with few addressing protection level calculations. Robust modeling is highlighted as a promising integrity method, combining quantification and qualification to address critical gaps. This approach offers a unified framework for improving localization system reliability and safety. This survey provides key insights for developing more robust localization systems, contributing to safer and more efficient autonomous operations.

## 1. Introduction

Integrity is a critical evaluation criterion for localization systems. It complements traditional performance metrics such as accuracy, availability, and reliability [1,2]. According to the **Federal Radionavigation Plan** [3], integrity is defined as follows: *“The measure of the trust that can be placed in the correctness of the information supplied by a positioning, navigation, and timing (PNT) system. Integrity includes the ability of the system to provide timely warnings to users when the system should not be used for navigation”.*

This definition highlights two key aspects of integrity: system trustworthiness and its ability to warn users of potential discrepancies. These aspects are particularly vital for high-stakes applications such as aviation and autonomous driving systems (ADS), where localization errors can have catastrophic consequences.

While this definition provides a solid foundation, new advances in localization technologies and increasing safety demands require a refinement of integrity concepts. High levels of automation, as defined by SAE [4], depend on accurate localization and guaranteed integrity. This paper explores revised definitions and methods for integrity, as discussed in Section 4.

Localization systems are affected by various error types (Section 2.1). Methods to handle these errors include fault detection and exclusion (FDE) (Section 6) and error quantification through the Protection Level (PL) (Section 5).

The PL *“represents an upper bound on the localization error”* [5]. It is widely used to indicate the error level in system estimates and remains one of the most prominent integrity metrics in the literature.

Integrity is critical for safety. In the United States alone, motor vehicle crashes cause over 40,000 deaths and 2 million injuries annually [6]. Localization errors can result in incorrect navigation decisions, leading to accidents [7]. Integrity also fosters consumer confidence, essential for the widespread adoption of autonomous vehicles [8]. It assures users of system reliability and enhances efficiency in navigation, route planning, and control, especially in challenging environments such as extreme weather or limited vision.

This survey focuses on integrity challenges related to sensors and algorithms in robotic systems, including autonomous vehicles. Sensor and algorithm failures compromise localization integrity. *Where no single method can address all faults and/or error types*. Hence, multiple techniques have been developed (Section 6 and Section 7).

Several existing surveys focus primarily on integrity monitoring (IM) for Global Navigation Satellite Systems (GNSSs). For example, ref. [9] discusses GNSS IM techniques, including Receiver Autonomous Integrity Monitoring (RAIM), fault detection, exclusion methods, and PL computation. Similarly, ref. [10] addresses GNSS-based IM for urban transport applications, noting challenges and open research areas compared to aviation.

Other reviews, such as [11], explore IM methods for GNSSs, INS, map-assisted, and wireless-augmented systems. It covers measurement errors, faults from various data sources, and the integration of sensors with GNSSs to improve navigation reliability. The review identifies challenges and highlights the need for advances in fault detection, exclusion, error modeling, and real-time processing. It also explores IM techniques for GNSS/INS with map-matching, discussing map/map-matching error handling and map constraints.

In contrast, our survey covers a wider range of localization methods. These include Simultaneous Localization and Mapping (SLAM), fusion-based, and optimization-based approaches. We address integrity challenges for diverse sensors such as LiDAR, cameras, HD maps, and INS. Our review emphasizes integrity in perception-based localization systems and highlights gaps in PL methods for these sensors.

**Figure 1:**
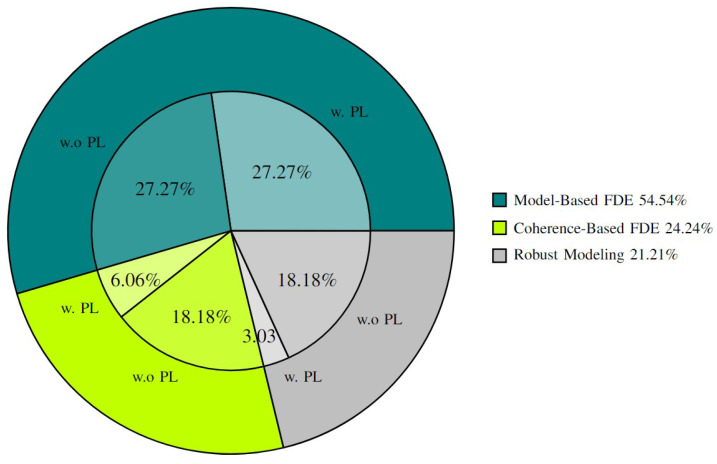
Percentage of surveyed literature on integrity methods, categorized as being w. (with) PL and w.o (without) PL.

Based on data from [9,10,12,13,14,15,16], 73.3% of the surveyed papers focus on GNSS-based integrity methods, while only 26.7% explore non-GNSS approaches. This imbalance highlights a research gap in perception-based localization systems (Section 5, Section 6 and Section 7). Figure 1 shows the distribution of surveyed papers and reveals the need for further exploration in PL methods for these systems.

**Figure 2:**
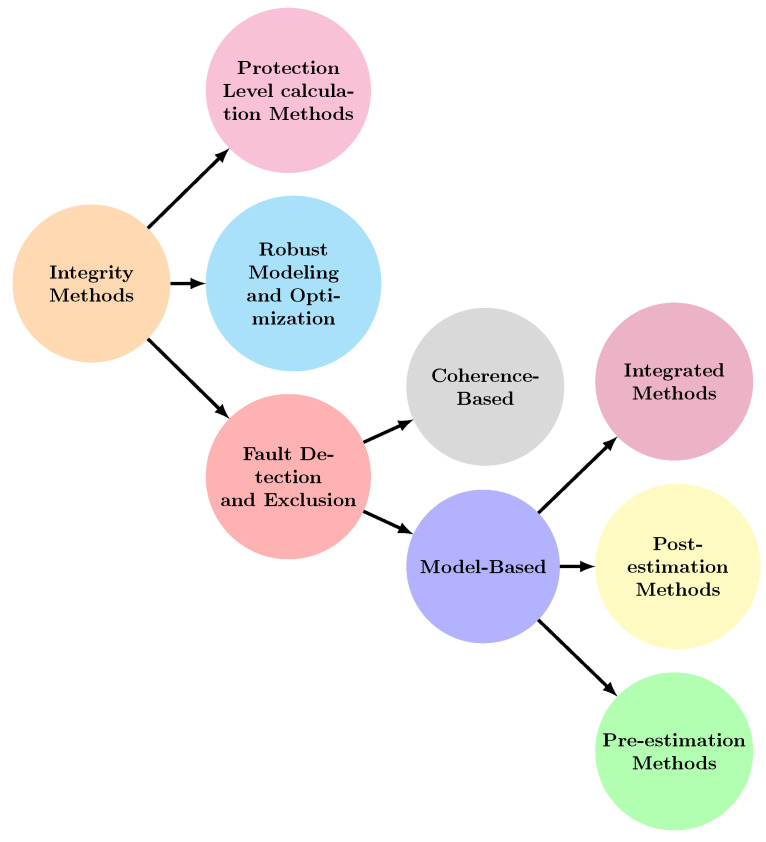
Classification of integrity methods.

To address this gap and advance the field, this paper makes the following contributions:

1. **Overview of integrity methods:** A thorough review of integrity methods for localization systems, covering sensors like LiDAR, cameras, HD maps, and INS.
2. **A new classification framework:** Introduction of a new categorization of integrity methods (Figure 2).
3. **Refined definitions:** Updated definitions of integrity and PL specific to localization systems, clarifying key concepts and metrics.
4. **In-depth review and comparative analysis:** A detailed analysis of robust modeling, PL computation techniques, and FDE methods.
5. **Detailed comparisons:** Comparisons of techniques, metrics, data types, sensors, and integrity enhancements.

In conclusion, integrity is a critical aspect of localization systems across various technologies, not just GNSSs. Existing research shows a lack of focus on PL methods for perception-based systems. This paper addresses this gap by providing a comprehensive framework, redefining integrity concepts, and advancing the understanding of integrity methods in localization systems.

The remainder of this paper is organized as follows. In Section 2, we introduce the key concepts related to error types and protection level parameters, providing a self-contained foundation for the subsequent sections. Section 3 offers a brief review of GNSS-IM systems, summarizing their relevance in localization frameworks. In Section 4, we revisit and discuss various integrity definitions proposed in the literature, concluding with our proposed integrity definition, which better aligns with the objectives of this work. Similarly, Section 5 reviews and analyzes existing definitions of the protection level and culminates with our proposed definition that addresses identified limitations. Section 6 introduces fault detection and exclusion methods, categorizing them into various approaches and subcategories to highlight their roles in ensuring robust localization. Lastly, Section 7 focuses on robust modeling and optimization techniques, presenting their qualitative and quantitative aspects and demonstrating their importance in enhancing localization system performance. Together, these sections aim to provide a comprehensive understanding of integrity and robustness within localization systems while presenting our contributions and findings.

## 2. Background and Foundational Concepts

This section defines the vocabulary used to describe the integrity of localization systems. First of all, this section introduces various error types that localization systems encounter. Then, the different perspectives on the integrity definition that appear in the literature are discussed. Finally the section concludes with a proposed integrity definition that encompasses the different dimensions of the definitions found in the literature.

We propose the following concepts and terms to make the whole discussion self-contained and easy to follow. These terms will help newcomers to the topic of integrity and localization in general to understand the concepts more easily and clearly.

### 2.1. Error Types

**Figure 3:**
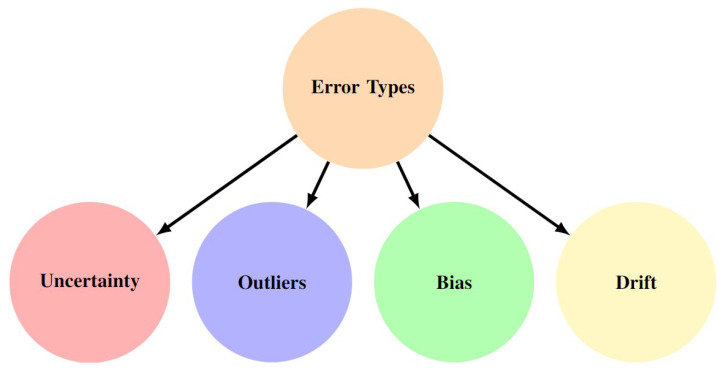
Classification of error types.

A localization system can be affected by various error types. These can significantly compromise the integrity of the system. The error types can be classified into four main categories: *uncertainty*, *bias*, *drift*, and *outliers*, as illustrated in Figure 3.

Each of these error types has *distinct characteristics and impacts* on the system’s performance, and understanding them is crucial for developing methods to improve localization integrity.

**Figure 4:**
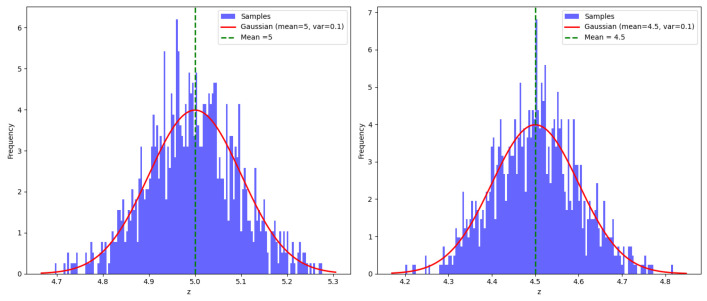
(**Left**): Histogram of LiDAR measurements at the true distance (5 m). (**Right**): Histogram of LiDAR measurements for the same true distance (5 m) with b=0.5 m bias error (mean = 4.5 m).

**Uncertainty,** often referred to as random error, is “a short-term scattering of values around a mean value” [17]. This type of error can be expressed using a probabilistic density function, such as the Gaussian distribution. It is depicted in the left image of Figure 4, where the measurement errors are distributed symmetrically around the true value, indicating random fluctuations over time.

**Bias,** or systematic error, is “a permanent deflection in the same direction from the true value” [17]. Unlike uncertainty, bias is not random but consistently skews measurements in one direction. The right image in Figure 4 shows a histogram of LiDAR measurements, where a bias of b=−0.5 m is added to the true value of 5 m, resulting in a shift in the mean of the measurement distribution.

**Figure 5:**
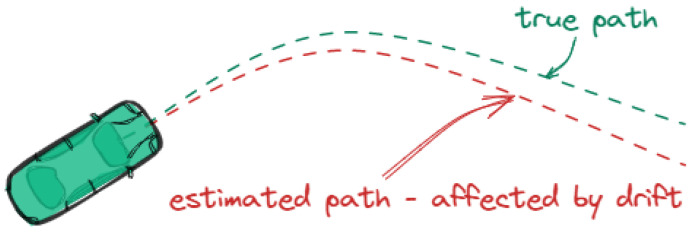
Due to the drift error, the vehicle’s estimated path is constantly deviating from the true path.

**Drift** refers to “errors that grow slowly over time” [11], often due to cumulative sensor inaccuracies or environmental factors. These errors can cause the estimated trajectory of a vehicle to deviate progressively from the true path, as shown in Figure 5. The figure demonstrates how the estimated path, affected by drift, diverges from the true path as time progresses.

**Figure 6:**
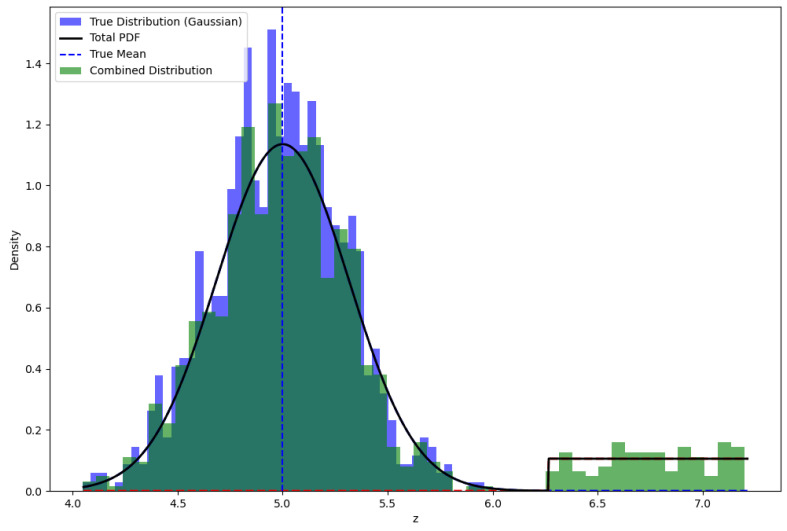
The true and outlier distributions along with their combined PDF. The true distribution (blue) is a Gaussian with mean 5 and variance 0.1. Outlier distribution (green) is a uniform PDF, shifted to the right. Probability of encountering an outlier δ is set to 0.1.

**Outlier** is “an observation that deviates so much from other observations as to arouse suspicions that it was generated by a different mechanism” [18]. This indicates that they might have been generated by a different mechanism. Outliers can occur due to sensor malfunctions, environmental disturbances, or other external factors that influence the measurement process. In the context of a LiDAR point cloud observation, an outlier is considered to be any data point that does not belong to the assumed population of true measurements. The presence of outliers is illustrated in Figure 6, where a uniform distribution of outlier values is combined with the Gaussian distribution of true values.

We adopt the generative models used in [18,19], which utilize a generic observation model to characterize the sensor behavior. We use a LiDAR sensor as an example. Each measurement, like a single LiDAR beam, is independent given the sensor’s pose. This assumption is important for the model’s simplicity and efficiency. It lets us model each beam’s measurement separately and then combine them to create a complete sensor model.

Consider a point cloud data observation from a LiDAR at time *t*, $Z_{t}=\{z_{t}^{1},z_{t}^{2},\ldots ,z_{t}^{k}$, $\ldots ,z_{t}^{N}\}$ , where *N* is the number of LiDAR beams. This observed point cloud does not contain only the true point cloud data, ${\bar{Z}}_{t}=\left\{{\bar{z}}_{t}^{1},{\bar{z}}_{t}^{2},\ldots ,{\bar{z}}_{t}^{k},\ldots ,{\bar{z}}_{t}^{N}\right\}$, but also different error types.

Starting with a single LiDAR beam, the measurement at time *t*, denoted as $z_{t}^{k}$, can be written as

(1) $$\begin{array}{c} z_{t}^{k}=\underset{\mathrm{true}\;\mathrm{value}}{\underset{︸}{{\bar{z}}_{t}^{k}}}+\underset{\mathrm{bias}\;\mathrm{value}}{\underset{︸}{b}}+\underset{\mathrm{drift}\;\mathrm{value}}{\underset{︸}{d(t)}}+\underset{\mathrm{uncertainty}}{\underset{︸}{\nu }} \end{array}$$

Here, $z_{t}^{k}$ represents the raw measurement from the LiDAR sensor, which consists of several components:

- The true value ${\bar{z}}_{t}^{k}$, which is the actual distance to the target;
- The bias b, which represents systematic errors that shift the measurement consistently [20,21,22,23];
- The drift d(t), representing errors that change over time, typically due to sensor aging or environmental influences;
- The uncertainty ν, which accounts for random noise in the measurement process.

Outliers, which are measurements that fall far outside the expected range of values, are accounted for separately in the model. In particular, the outlier distribution is modeled using a combined probability density function (PDF), which accounts for both the true measurements and the outliers. The probability of encountering an outlier is represented by δ, and the combined PDF for a LiDAR measurement is expressed as

(2) $$p\left(z_{t}^{k}\right)=\left(1-\delta \right)\;p_{basic}\left(z_{t}^{k}\right)+\delta \;p_{outlier}\left(z_{t}^{k}\right)$$

This equation combines the likelihood of the measurement being a true value with the likelihood of it being an outlier. Here,

- $p_{basic}\left(z_{t}^{k}\right)$ is the PDF for an observation, which is a PDF of the random variable $z_{t}^{k}$ in Equation (1);
- $p_{outlier}\left(z_{t}^{k}\right)$ is outlier PDF.

The combined PDF allows us to model the probability that each measurement is either a true value or an outlier, which is important for robust localization in the presence of sensor errors.

Introducing more information about outliers into the model will *improve its ability to handle and account for them*. This leads to more accurate results. The model’s outlier handling mechanism is crucial. It ensures that the localization system can handle erroneous data points and provide accurate estimates, even with sensor faults or disturbances.

However, **no single method can handle all these error types and remove their effect on the final estimate**. Different methods are required for this, which are discussed in Section 6 and Section 7.

### 2.2. Protection Level Related Parameters

To facilitate the understanding of the protection level concept, several related terms and vocabulary are essential. The **position error**, denoted as *E*, represents the difference between the estimated pose, *x*, and the true pose, $x_{true}$, i.e., $E=||x-x_{true}\left|\right|$. In systems like RTK-GNSS, the true pose is estimated accurately, yet the localization system often lacks knowledge of the true path. The position error is typically modeled by a probability distribution, P(E), which can be influenced by various error types, Section 2.1, and approximations. In an ideal scenario, assuming a linear system, Gaussian distributions, and the absence of bias, drift, and outliers, position error would be normally distributed, i.e., $E∼N(0,\Sigma_{E})$.

**Accuracy** is defined as the degree to which the estimated position of the system approaches the actual position, as described by [10,11]. The **Alarm Limit** (AL) “*represents the largest position error allowable for safe operation*” [10]. When the error exceeds the AL, the localization system is deemed unsafe to rely on.

**Integrity risk** (IR) is the probability that the position error, *E*, exceeds the AL, E>AL, which can be expressed as

(3) $$IR_{AL}=P\left(E>AL\right)=\int_{AL}^{-\infty }P\left(E=e\right)de$$

However, since the AL can change over time and in different contexts, as noted in [24], the PL is used as a more stable alternative. This leads to an updated definition of IR in terms of PL, as presented in [25,26,27]:

(4) $$IR_{PL}=P\left(E>PL\right)=\int_{PL}^{-\infty }P\left(E=e\right)de$$

IR can be intuitively understood as the likelihood that the position error exceeds the AL, typically quantified per hour or per mile. This probability reflects the likelihood of undetected failures within the system that may lead to inaccurate or unsafe pose estimates. It is crucial for evaluating the robustness of localization systems, particularly in critical applications where safety is paramount.

**Target Integrity Risk** (TIR) is defined as the maximum acceptable level of IR, essentially an upper bound on IR [25,26,27]. This threshold is determined based on industry standards and safety requirements, and it ensures that the localization system remains safe within specified operational contexts. The IR must be continuously monitored to ensure that the system operates within acceptable limits. Additionally, the IR is calculated using system performance data, error models, and the prevailing operating conditions. The relationship between IR and TIR can be expressed as

(5) $$IR_{PL}<TIR$$

**Figure 7:**
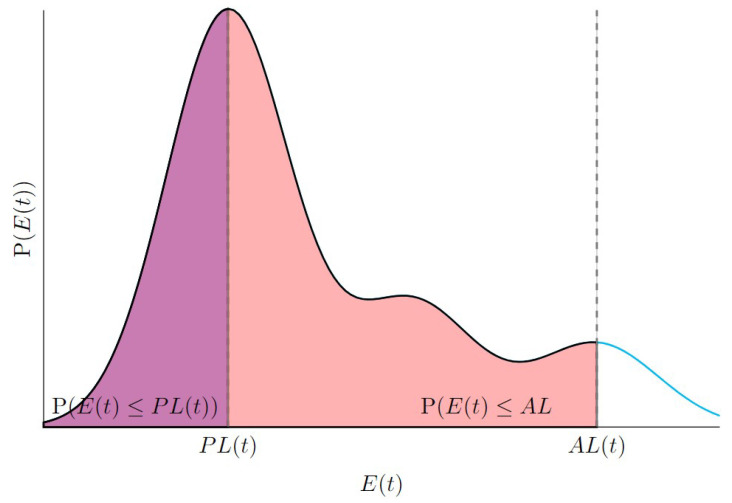
An arbitrary error distribution for a localization system at time *t*. The shaded regions indicate the probability that the error E(t)=e falls within those areas.

Figure 7 shows how the PL is evaluated at a specific time *t*, given an arbitrary error distribution. For a given PL, we can compute the probability that the error is below this level, based on its probability distribution at time *t*. Similarly, for a given AL, we can determine the probability that the error is below the AL. This allows us to calculate the IR for the PL.

This is the forward approach. We use a given PL to check if it satisfies the integrity risk criterion. However, the main goal is to find the PL that ensures a specified integrity risk for a given context and period. This is performed based on the error distribution at that time. Thus, we aim to determine the PL that guarantees the desired level of integrity risk.

Usually, TIR is used to refine and adjust the PL by tuning its parameters to fit the entire trajectory of the localization system. This is typically performed offline, using a learning approach with training and validation datasets, as described in [26]. The goal is to find the parameters that best fit the whole trajectory. In contrast, our focus is on estimating the PL in real time at each time step based on the current error distribution for a given IR.

## 3. Integrity Methods in GNSSs

Understanding integrity in localization systems is essential for many applications. Global Navigation Satellite Systems (GNSSs) provide the foundational methods for achieving integrity in localization. Building upon this foundation, this paper focuses on **extending** these integrity concepts to *perception-based* localization systems. This section presents key integrity methods used in GNSSs. It highlights fundamental techniques and concepts. **The overview is not exhaustive**. It does not cover all aspects of GNSS integrity. For a more detailed review, readers are encouraged to consult the surveys [9,10].

GNSSs use various integrity methods, mainly categorized into **Receiver Autonomous Integrity Monitoring** (**RAIM**) and (**PL**).

**RAIM** checks the consistency of multiple satellite signals by computing the position using different subsets of satellites and comparing the results. It requires a minimum of five satellites to detect faults. However, it is typically limited to handling only one faulty satellite at a time.

**Figure 8:**
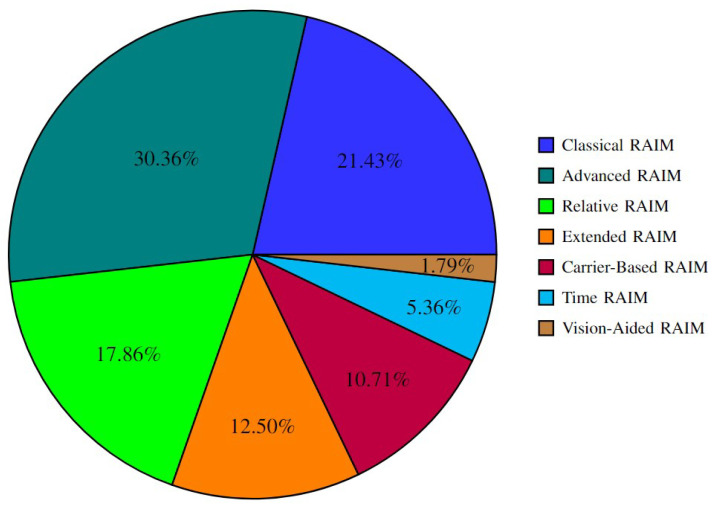
RAIM method classification.

Different RAIM variants use various measurements, like code or carrier measurements. They also differ in their fault detection capabilities. Advanced RAIM, for instance, can handle multiple faults more effectively compared to traditional RAIM. For a detailed comparison of measurement types and fault detection capabilities in these variants, refer to the surveys [9,10]. In this section, the results from these surveys are summarized and illustrated in Figure 8, with a focus on the RAIM variants.

PL depends on satellite-user geometry and expected pseudorange error. For example, in SBAS (Satellite-Based Augmentation System), PL is calculated as [28]:

(6) PL=Kσ

where *K* is an inflation constant and σ represents the confidence in the estimated position, measured in meters. Accurate PL computation requires knowledge of the distribution of residual position or range errors [29,30,31]. While PL has several formal definitions, discussed in Section 5, this informal description captures its essence and primary use in GNSSs.

## 4. Revisiting Integrity: Review, Enhancement, and New Definition

In this section, various definitions of “*integrity*” as presented in the literature on localization systems are reviewed and analyzed. Each definition’s approach to integrity is examined, where strengths and limitations are highlighted. It is important to note that this paper revisits the definitions of integrity, primarily in the context of non-GNSS localization systems. We begin by presenting the standard definition used in GNSS-based systems, such as the one outlined in the **Federal Radionavigation Plan**, and then analyze the integrity definitions in non-GNSSs that rely on perception sensors, such as LiDAR and cameras. This analysis seeks to complement and expand upon the existing literature in this area.

Tossaint et al. (2007) [32] define integrity for GNSS-based localization as “*the system’s ability to provide warnings to the user when the system is not available for a specific operation*”. Similarly, the Federal Radionavigation Plan defines integrity as “*the measure of the trust that can be placed in the correctness of the information supplied by a positioning, navigation, and timing (PNT) system. Integrity includes the ability of the system to provide timely warnings to users when the system should not be used for navigation*”. While these definitions emphasize providing warnings, they rely heavily on the context of specific user applications and operational requirements. The concept of integrity is reduced to the system’s ability to issue warnings, which, as a standalone metric, is vague and insufficient. Furthermore, neither definition provides a framework to quantify terms like “correctness” or “trust,” which are central to a robust understanding of integrity.

Ochieng et al. (2003) [33] and Larson (2010) [34] similarly define integrity as “*the navigation system’s ability to provide timely and valid warnings to users when the system must not be used for the intended operation or phase of flight*”. However, their definitions focus only on giving a warning but don’t explain much about what the problem is or how serious it could be. That’s why we need definitions that not only give warnings but also provide clear and useful (actionable) information.

Li et al. (2019, 2020) [35,36] describe integrity as “*the degree of trust that can be placed on the correctness of the localization solution, and compared it with the 3σ used in visual navigation*”. While this perspective provides a quantitative approach, it is unclear whether the comparison to 3σ is limited to vision-based systems or can be generalized to other localization approaches. This lack of clarity reduces its applicability across different system types.

In contrast, AlHage et al. (2021, 2022, 2023) [25,26,27] define integrity as “*the ability to estimate error bounds in order to address uncertainty in the localization estimates in real time.*” This definition shifts the focus toward quantifying uncertainty through error bounds, emphasizing that these bounds should include the true position. However, their work implicitly incorporates FDE methods, referred to as “internal integrity,” to enhance the system’s overall performance. This implicit connection is not clearly articulated in their definition, which could lead to ambiguity.

Arjun et al. (2020) [37] define integrity as “*the measures of overall accuracy and consistency of data sources.*” Although this definition highlights data source consistency, it does not address fault impacts or provide mechanisms for evaluating how faults affect system integrity quantitatively. As such, it lacks the necessary depth for a comprehensive understanding of system performance.

Bader et al. (2017) [38] describe integrity as “*the absence of improper system alterations*”. This definition adopts a rigid view, equating any fault with a complete loss of integrity. By failing to distinguish between the varying impacts of different faults, this perspective oversimplifies the concept. A more nuanced approach would involve quantifying fault impacts to better evaluate system integrity.

Wang et al. (2022) [39] describe integrity as “*an important indicator for ensuring the driving safety of vehicles*”. However, this definition lacks specificity, as it does not elaborate on how integrity relates to localization systems or explain its role in ensuring safety.

Quddus et al. (2006) [40] focus on a narrower context, defining integrity as “*the degree of trust that can be placed in the information provided by the map matching algorithm for each position.*” This definition restricts the scope of integrity to map matching algorithms, ignoring other critical components of localization systems.

Marchand et al. (2010) [41,42], Sriramya (2021) [43], and Shubh (2023) [44] describe integrity as “*the measure of trust which can be placed in the correctness of the information supplied by the total system*”, “*the measure of trust that can be placed in the correctness of the estimated position by the navigation system*”, and “*the measure of trust that can be placed in the accuracy of the information supplied by the navigation system*”, respectively. While these definitions focus on trust and correctness, they fail to frame integrity as an evaluation criterion for the entire localization system. Vague terms like “correctness” and “trust” are left undefined, creating gaps in understanding how they relate to assessing system performance. This oversight also neglects the broader need to evaluate how well the system manages errors and deviations, which is essential for a comprehensive assessment of its integrity.

The reviewed definitions of integrity provide valuable insights but reveal several critical gaps that necessitate a more comprehensive framework:

- **Overemphasis on warnings:** Existing definitions, such as those by Tossaint et al. [32], Ochieng et al. [33], and Larson [34], focus heavily on issuing warnings without addressing broader aspects like error management or quantification.
- **Lack of quantification:** Terms like “trust” and “correctness,” central to definitions by Tossaint et al. [32], Marchand et al. [41], and others, are vague and unmeasurable, limiting their practical applicability.
- **Limited scope:** Definitions such as Quddus et al.’s [40] focus narrowly on specific components (e.g., map matching) rather than the entire localization system.
- **Fault impact and error management:** Works like AlHage et al. [25,27] implicitly address FDE but fail to clearly articulate its connection to integrity as a measurable concept.
- **Oversimplification:** Definitions like Bader et al.’s [38] equate any fault with a complete loss of integrity, oversimplifying the varied impacts of different fault types.
- **Misalignment with real-time systems:** While some works, such as AlHage et al. [27], propose real-time error estimation, they lack clarity in connecting these estimates to actionable metrics like PL.
- **Insufficient robustness considerations:** Few definitions explicitly address robustness or outlier handling, a crucial aspect of real-world localization systems.

**This paper addresses these gaps by proposing a new definition of integrity that combines both qualitative and quantitative dimensions**. Integrity is redefined as the system’s alignment with reality, encompassing robustness, outlier handling, and deviation measurements. The proposed definition:

**Definition** **1** (Integrity)**.**  *Integrity refers to the quality of a system being coherent with reality.*

**Definition** **2** (Integrity for Localization Systems)**.**  *In the context of a localization system, integrity serves as an important evaluation criterion, encompassing both **qualifying** and **quantifying** aspects:*

- ***Qualifying aspect*** *: Integrity represents the system’s ability to remain unaltered and effectively handle outliers and errors;*
- ***Quantifying aspect*** *: Integrity also involves providing an overbounding measure of **how far** the system’s outputs can deviate from reality.*

*The term **how far** will be formally quantified in Section 5.*

The proposed framework evaluates robustness and reliability comprehensively. Qualitative methods focus on ensuring system reliability and managing outliers effectively, while FDE methods (Section 6.1 and Section 6.2) play a key role in mitigating errors. Quantitative methods, like PL (Section 5), measure deviations between the system’s outputs and reality.

New robust modeling and optimization techniques, discussed in Section 7 and illustrated in Figure 2, enhance the system’s ability to handle outliers. These techniques provide probabilistic interpretations of errors, improving the assessment of localization systems.

The paper reviews integrity methods across various localization systems and sensors. It also redefines PL as a core metric for quantifying integrity, aligning it with the proposed definition.

## 5. Protection Level: Current Definitions and New Perspectives

In the following discussion, multiple definitions of PL found in the literature will be outlined. These definitions capture various meanings and applications of PL in the context of integrity for localization systems. Following this review, a proposed definition of PL will be presented to broaden and enhance our understanding of this crucial topic.

Li et al. (2019, 2020) [35,36] describe PL as “*the highest translational error resulting from an outlier that outlier detection systems cannot detect*”. This definition is limited to translational errors and does not fully address how PL should encompass all types of uncertainties, including those from various sources beyond undetected outliers. Moreover, it fails to capture the complete error region within which the true position is guaranteed and does not consider the full scope of errors from all system components and algorithms.

Marchand et al. (2010) [41,42] define PL as “*the result of a single undiscovered fault on the positioning error*”. Similar to the previous definition, this one is confined to undetected faults and does not account for multiple faults or the broader uncertainty inherent in sensor measurements.

The importance of PL as “*a statistical bound on position error, E, that guarantees that IR does not exceed TIR*” is highlighted by AlHage et al. (2021, 2022, 2023) [25,26,27]. In a similar way, Arjun et al. (2020) [37] and Sriramya (2021) [43] define PL as “*an error bound linked to a pre-defined risk*”. While these definitions connect PL to localization system requirements, where TIR is used to check for undetected faults, they do not fully address how PL should account for all uncertainties from various system components.

Shubh (2023) [44] defines PL as “*the range within which the true position lies with a high degree of confidence*”, while Wang et al. (2022) [39] describe it as “a*n upper bound on positioning error*”. Larson (2010) [34] states PL as “*ensuring that position errors remain within allowable boundaries, even with faults*”. While Wang’s use of “upper bound” is overly general, Larson’s focus on error boundaries relates more to system error minimization and handling and does not clearly separate PL from the system’s accuracy.

Overall, current definitions of PL provide useful insights but reveal several critical gaps:

- **Limited scope of errors considered:** Definitions by Li et al. [35,36] and Marchand et al. [41,42] focus narrowly on undetected faults, failing to account for multiple simultaneous faults or uncertainties from system components, such as sensor noise or environmental factors.
- **Lack of comprehensive uncertainty coverage:** While definitions by AlHage et al. [25,26,27] and Sriramya [43] connect PL to statistical bounds, they do not fully encompass uncertainties from all sources, including sensor noise, dynamic conditions, and processing errors.
- **Generalization without specificity:** Definitions by Wang et al. [39] and Larson [34] use vague terms like “upper bound”, which fail to distinguish PL as a distinct metric from accuracy or precision.
- **Inconsistent real-time relevance:** Shubh’s [44] focus on confidence lacks a connection to real-time adaptability, which is crucial for ensuring integrity in dynamic environments.
- **Separation from integrity assessment:** Many definitions fail to explicitly link PL as a core metric for evaluating and maintaining system integrity, limiting their practical applicability.

To address these gaps, the proposed definition of PL is

**Definition** **3** (Protection Level)**.** *Protection Level is the real-time estimate or calculation of the error region within which the true position is guaranteed to lie.*

By assigning PL to each state estimate, the localization system can effectively adapt to changing environments, sensor conditions, and vehicle dynamics. As a result, system integrity is properly assessed and maintained in real time.

## 6. Fault Detection and Exclusion

FDE is crucial for enhancing the integrity of the localization system. FDE ensures accurate outputs despite faults or deviations in sensors or algorithms intended behavior. It works by identifying and removing faulty data. “Faults”, “failures”, and “outliers” often refer to deviations that can negatively impact estimation accuracy. The FDE process addresses the qualifying aspect of integrity by making the localization resilient to various error types.

The literature distinguishes between FDE and Fault Detection and Isolation (FDI). FDE focuses on detecting and excluding anomalies to maintain integrity, **without identifying the specific error types that caused the deviation from the true value**. However, FDI aims to identify the specific cause of the problem, which is more relevant in the control engineering and software industries. For localization systems, the key objective is detecting and excluding abnormalities, regardless of their cause. Therefore, this discussion considers all approaches under the category of FDE.

**Figure 9:**
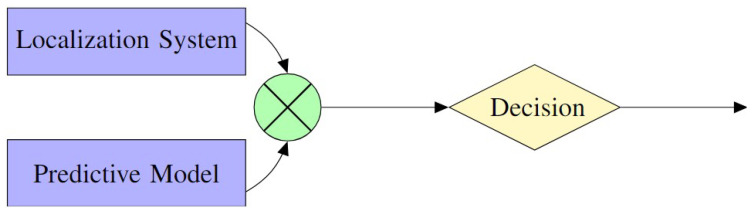
Model-based fault detection and exclusion: A schematic representation illustrating the integration of predictive models for system behavior and localization systems.

**Figure 10:**
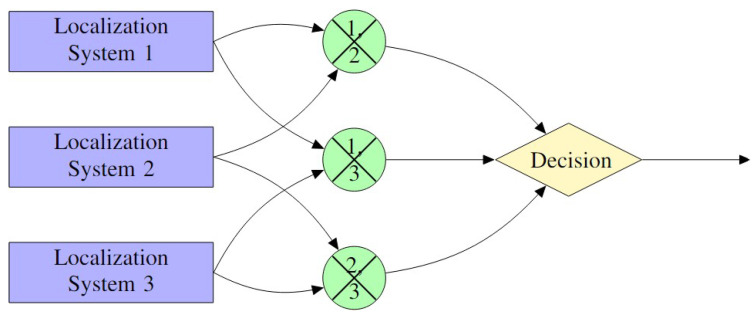
Coherence-based fault detection and exclusion: The figure illustrates three localization systems (1, 2, and 3) undergoing coherence checks. Comparative analyses are performed between systems 1 and 2, 2 and 3, and 1 and 3 to identify any non-coherent behavior.

Extensive literature analysis reveals two possible main categories of FDE techniques: model-based and coherence-based approaches. Model-based techniques, Figure 9, utilize mathematical models to predict the system’s behavior, identifying deviations as potential faults. Coherence-based techniques, Figure 10, leverage the consistency among various sensors or measurements of the same quantity, flagging incoherent data points as potential faults.

The following sections explore each category in detail, including methods for computing the protection level. We will use some illustrative figures from the reviewed references. Not all figures will be included; only those that help clarify the process will be selected.

### 6.1. Model-Based FDE

In the field of FDE in localization and navigation systems, Model-Based FDE, or MB-FDE, is a vital component, providing reliable solutions through the use of predictive models of system behavior; see Figure 9. These predictive models could be sensor models, system models, or machine learning models like Convolutional Neural Networks (CNNs).

MB-FDE techniques identify discrepancies between expected and observed values to detect and exclude faulty data. MB-FDE techniques identify faults by analyzing discrepancies between predicted and observed values.

As an illustrative example following Figure 9, the mathematical derivation involves calculating the residuals r(t). These residuals represent the difference between the predicted output $\hat{y}\left(t\right)$ and the observed output y(t):

(7) $$r\left(t\right)=y\left(t\right)-\hat{y}\left(t\right)$$

where

- r(t) is the residual at time *t*;
- y(t) is the observed value at time *t*;
- $\hat{y}\left(t\right)$ is the predicted value based on the system model.

To determine if the discrepancy is significant enough to indicate a fault, the residuals are compared against a threshold. This threshold is typically derived from the statistical properties of the residuals, often using a Chi-square test. The Chi-square statistic quantifies the discrepancy between the residual vector and its expected distribution:

(8) $$\chi^{2}=r{\left(t\right)}^{T}R^{-1}r\left(t\right)$$

where

- r(t) is the residual vector at time *t*;
- R is the covariance matrix of the residuals, which models the expected variability of the residuals under normal operating conditions.

For a properly functioning system, the Chi-square statistic follows a known distribution. The threshold $\gamma_{\alpha }$ is selected based on a desired confidence level, 1−α, where α is the significance level. This corresponds to a critical value from the Chi-square distribution with appropriate degrees of freedom, typically the number of residuals.

If the calculated Chi-square statistic exceeds this critical value, the discrepancy is deemed too large to have occurred under normal conditions, indicating a fault. Then, the fault exclusion rule is

$$\gamma_{\alpha }=\chi_{\alpha ,m}^{2}$$

If the test statistic satisfies $\chi^{2}>\gamma_{\alpha }$, the measurement is flagged as faulty and excluded from the estimation process. Otherwise, the measurement is considered valid:

$$\chi^{2}>\gamma_{\alpha }\;\Rightarrow \;\mathrm{Fault}\;\mathrm{Detected}$$

By dynamically generating the threshold based on the statistical properties of the residuals, this method ensures that the system remains robust to normal variations while being sensitive enough to detect faults. The previous example illustrates the general framework of the MB-FDE methodology. As will be illustrated in the following sections, variations in approaches within this domain arise primarily from differences in the computation of residuals and the selection of thresholds, which are often based on specific statistical distributions. The localization algorithm and input number and type affect these variations.

In the process of this review, a wide range of techniques will be examined, each of which will provide special insights for improving the integrity and PL calculation.

Based on the surveyed papers, MB-FDE is further categorized into three types:

1. Post-estimation MB-FDE (Section 6.1.1);
2. Pre-estimation MB-FDE (Section 6.1.2);
3. Integrated (or Embedded) MB-FDE (Section 6.1.3).

Table 1, Table 2 and Table 3 provides a summary of all MB-FDE methods, comparing them across various criteria. The first two tables focus on ground vehicles, while the last table covers multi-ground vehicles and micro aerial vehicles. Each of these categories will be explained in the following sections.

#### 6.1.1. Post-Estimation MB-FDE

**Figure 11:**
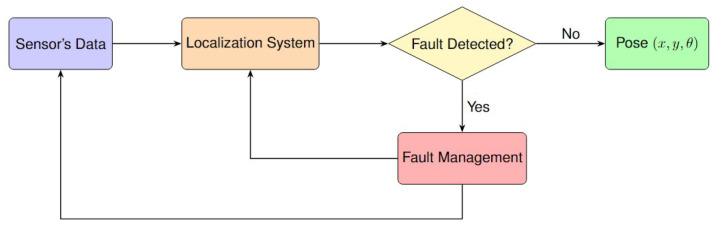
Post-estimation FDE process in localization systems.

In the post-estimation scheme, FDE is applied after the localization system has produced an estimate, such as the pose. First, the localization algorithm performs data fusion, Bayesian updates, or optimization. Then, faults or outliers are detected. This means measurements are used as they are, known as the sensor level, and fault detection happens at the system level, like the state or pose. Therefore, detection of faults or outliers happens after the localization processing is complete; see Figure 11. The following provides an in-depth review of post-estimation FDE methods found in the literature.

In [25,26], an Extended Information Kalman Filter (EIF) is introduced. Banks of EIFs estimate the state using sensors like GNSS, cameras, and odometry. Each filter’s output is compared to a main filter that combines, fuses, all outputs. Residuals, calculated as the Mahalanobis distance, were used to identify and exclude deviant filters and their sensor data.

PL is calculated by over-bounding the EIF error covariance with a Student’s *t*-distribution. The degree of freedom for this distribution is adjusted offline during a training phase. However, this method has limitations. It assumes Gaussian noise, which doesn’t accurately represent noise during faults or outliers. The thresholding also depends on this assumption, using Mahalanobis distance compared to a Chi-square distribution. Additionally, the PL calculation is adjusted offline to find the best degree of freedom for the *t*-distribution for a specific trajectory or scenario.

**Figure 12:**
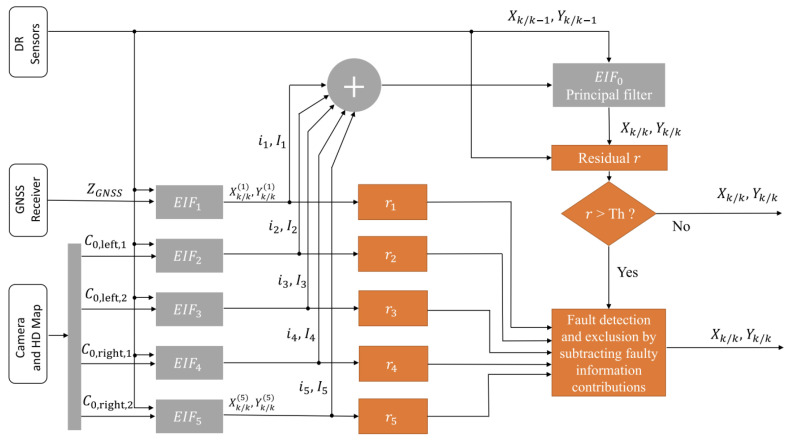
Multisensor fusion with FDE uses camera measurements and the HD map as inputs. In the context of the information filter for multisensor fusion: *Z* denotes observations from GNSS or cameras, X=(x,y,θ) is the state vector, *Y* is the information matrix, and *y* is the information vector. $I_{i,k}$ and $i_{i,k}$ represent the information contributions from observation $Z_{i}$. This figure was adopted from [26].

In [27], a Student *t*-distribution EIF (t-EIF) is utilized, akin to [25,26], but with different residual generation methods. Instead of Mahalanobis distance, it employs Kullback–Leibler Divergence (KLD) between updated and predicted distributions. Residual values adaptively adjust the *t*-distribution’s degree of freedom, enhancing robustness against outliers. Larger residuals indicate noisy measurements, necessitating thicker tails and lower degrees of freedom, while smaller residuals justify higher degrees of freedom. This adaptation is governed by a negative exponential model, ensuring flexibility and optimization for various measurement conditions. PL calculation depends on degrees of freedom at the prediction and update steps. Errors are adjusted based on the minimum degrees of freedom between these steps. The final PL formula mirrors that of [25,26]. Figure 12 shows a general diagram of how the EIF is applied for FDE.

A multirobot system with an FDE step is addressed in [59]. The approach utilizes an EIF-based multisensor fusion system. The Global Kullback–Leibler Divergence (GKLD) between the a priori and a posteriori distributions of the EIF is computed as a residual. This residual, dependent on mean and covariance matrices, is utilized to detect and exclude faults from the fusion process. First, the GKLD is used to detect faults. Next, an EIF bank is designed to exclude faulty observations. The Kullback–Leibler Criterion (KLC) is then used to optimize thresholds in order to achieve the optimal false alarm and detection probability.

In [60], a multirobot system uses EIF for localization. Each robot performs local fault detection based on its updated and predicted states. The Jensen-Shannon divergence (JSD) is applied to generate the residuals between distributions. Fault detection thresholds are set using the Youden index from ROC curves. When a fault is detected, JSD is computed for predictions versus corrections from Gyro, Marvelmind, and LiDAR. Residuals are categorized based on their sensitivity to different error types and errors from nearby vehicles. If thresholds are exceeded, residuals are activated using a signature matrix, which helps detect and exclude faults and detects simultaneous errors. Faulty measurements are then removed from the fusion process.

In [55,58], the setup is extended with a new FDE approach and batch covariance intersection informational filter (B-CIIF). Fault detection and exclusion are based on JSD between predicted and updated states from all sensors.

In [55], a decision tree is employed for fault detection, while a random forest classifier is used for fault exclusion. Both methods use JSD residuals and a prior probability of the no-fault hypothesis for training. In contrast, ref. [58] employs two Multi-layer Perceptron (MLP) models. One for fault detection and the other for fault exclusion. The input to the MLPs includes residuals and the prior probability of the no-fault hypothesis.

Training data for these machine learning techniques include various fault categories like gyroscope drift, encoder data accumulation, Marvelmind data bias, and LiDAR errors. However, the limitation of generalizability remains significant. Since the training data are specific to certain scenarios, the models may struggle to detect and address faults in new or unfamiliar environments. This limitation can compromise the overall reliability and integrity of the system when deployed beyond the scope of the training data.

#### 6.1.2. Pre-Estimation MB-FDE

**Figure 13:**
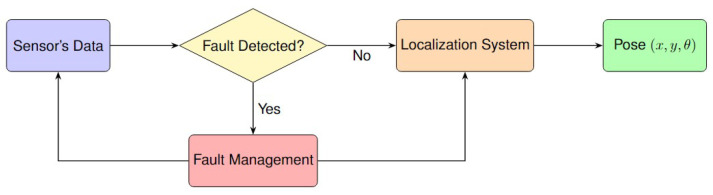
Pre-estimation FDE process in localization systems.

In the pre-estimation scheme, FDE is applied before the localization estimate is made at the sensor level. Sensor measurements, such as from LiDAR, cameras, odometry, or GNSS, are first checked for faults or outliers. This means faults are detected and excluded before the data are used in the localization system, whether it involves fusion, Bayesian updates, or optimization; see Figure 13. An in-depth review of pre-estimation FDE methods from the literature is presented next.

**Figure 14:**
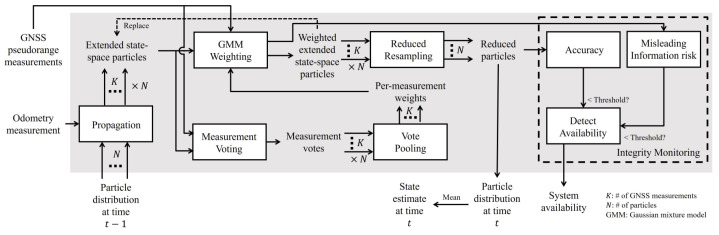
The framework addresses GNSS faults with GMM weighting with the voting scheme. This figure was adopted from [45].

In GNSSs, ref. [45] presents a method to reduce residuals between predicted and measured pseudo-range data from satellites. They use a Gaussian Mixture Model (GMM) to handle errors that have multiple modes. Instead of a single linearization point, they use a distribution over this point, managed by a particle filter. Each particle has a vector indicating the weight from each satellite. The particle with the highest total weight is favored; this is called the voting step. This method relies on data from multiple satellites and GNSS receiver correlations over time. If any data are missing, the method’s accuracy declines. Integrity is measured by calculating the likelihood that the estimated pose exceeds the AL region. Figure 14 illustrates the framework adopted in this work.

Integrity is assessed using two metrics: hazardous operation risk and accuracy. Accuracy measures the probability that the estimated pose is outside the AL, while hazardous operation risk examines if the estimated position has at least a 50% probability of containing the true position. An alarm is triggered if either metric exceeds a set threshold, indicating a loss of integrity.

In [35,36], a vision-based localization system enhances ORB-SLAM2 with FDE techniques. It uses a parity space test to detect faults, addressing one fault at a time by comparing expected and observed measurements. Faulty features are removed iteratively until the error hits a threshold. An adaptive residual error calculation accounts for uncertainties. The modular design allows easy integration with existing SLAM techniques. PL considers noise from observations and maximum deviation from undetected faults, calculated as a weighted sum of covariance elements. Improved covariance matrix elements boost computation accuracy by removing inaccurate features or outliers iteratively. A threshold ensures a sufficient number of inliers for SLAM operations; if not met, the location estimate is deemed unsafe.

The technique used for FDE in the image-based navigation system described in [34] is similar to that of GPS RAIM [61,62,63], which is based on performing a parity test for FDE as in [35,36]. PL is calculated based on the maximum slope of the worst-case failure model, similar to concepts discussed in [46,47]. The author of this work uses a feature-based tracking method for localization.

Refs [41,42] present a method to reduce errors by treating GNSS and odometry data separately. They improve state estimation by using trajectory monitoring and a short-term memory buffer instead of relying on the standard Markovian assumption. Their technique estimates states over a finite horizon and uses sensor residuals and variances to weight GNSS and odometry data.

For fault detection, they calculate sensor residuals, squared errors, and variances. They then weight GNSS and odometry data based on the ratio of each residual to its standard deviation. A Chi-square distribution threshold is used for detecting faults. The sensor with the highest weight is prioritized for exclusion.

This approach relies on hyperparameters for its operation. These include the misdetection probability for the protection level calculation and the Chi-square distribution for fault detection. Additionally, the size of the state buffer is a hyperparameter that should be selected based on the environment and scenario.

The technique presented in [39] is customized for FDE in real time within LiDAR mapping and odometry algorithms. This technique uses a feature-based sensor model to compute the mean and variance of the latest *k* innovations for the Extended Kalman Filter (EKF) setting. This technique adaptively establishes a threshold for fault detection by using the Chi-square distribution. Thus, the technique can adapt to environmental changes and sensor conditions. Feature-based FDE technique, dynamic thresholding, and real-time noise estimates are combined in this technique to effectively detect and exclude faults in LiDAR odometry and mapping algorithms. The technique lacks a particular formula for estimating the protection level. Its integrity is, however, evaluated by analyzing critical parameters, including error boundaries, missed detection rate, and false alarm rate. Among these parameters, the error bound stands out as a critical signal for integrity assessment, showing the maximum possible pose error.

In [48], each sensor’s output is evaluated using Hotelling’s $T^{2}$ test, based on expected sensor output and covariance. This allows for accurate fault detection by examining the correlation within the same sensor’s data. By employing the Student *t*-distribution to overbound measurement noise and measurement innovation sample covariance to inflate it, where measurement noise is adaptively updated. The adaptive updating of the measurement noise covariance is similar to [39]. It takes into account faults and other outliers adaptively. This application uses a UKF localization-based methodology.

**Figure 15:**
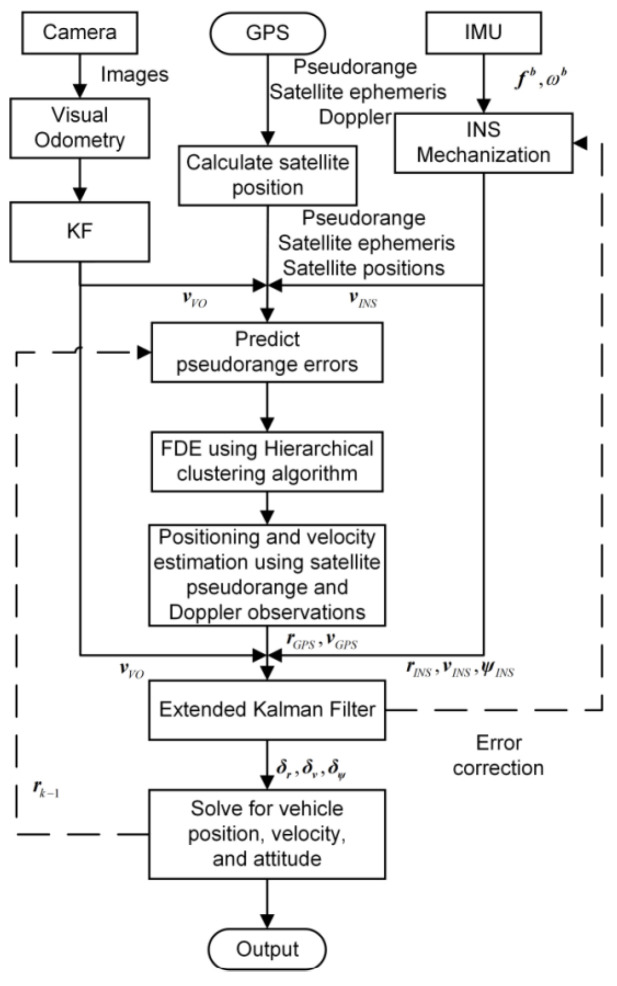
The algorithm framework of using hierarchical clustering for FDE. This figure was adopted from [54].

The method in [54] uses raw GNSS measurements for FDE; see Figure 15. Vehicle pose is predicted using IMU and Visual Odometry (VO). The error between this prediction and the GPS receiver pose estimate is computed. FDE is implemented using Hierarchical Clustering [64] to detect and exclude faulty satellite signals. Satellites are divided into three clusters based on estimated errors: multipath, Non-Line of Sight (NLOS), and Line of Sight (LOS) without errors. Initially, each satellite signal is considered a cluster, which is then clustered based on similarity. The three resulting clusters represent the main types of GPS errors. The LOS cluster, presumed to have the most samples, is used to compare expected pseudo-range errors. If this error exceeds a preset threshold, the associated satellite is excluded. The remaining measurements are used to calculate the GPS receiver’s position and velocity. However, selecting and adjusting thresholds lacks standardization, and the method to calculate the threshold in [54] is not specified.

#### 6.1.3. Integrated MB-FDE

**Figure 16:**
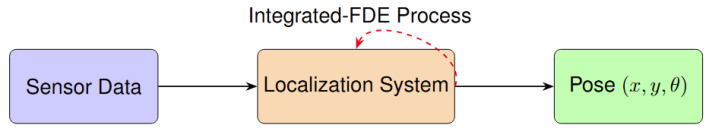
Integrated FDE process in localization systems.

In the integrated (or embedded) FDE scheme, FDE is incorporated within the localization process itself, suggesting that the fault detection and exclusion steps occur simultaneously with the localization algorithm, rather than as a separate phase. As sensor data are collected, they are immediately checked for faults or outliers, often using a weighting or selection method, before being used for localization. This means that the system continuously adjusts how each measurement affects the final output, such as the pose. For example, if a sensor reading is found to be unreliable, it is down-weighted or ignored during the data fusion or optimization steps. Unlike pre-estimation or post-estimation methods, which handle faults before or after main processing, integrated FDE handles faults dynamically as data are processed; see Figure 16. Next, a detailed examination of integrated FDE methods in the literature is provided.

**Figure 17:**
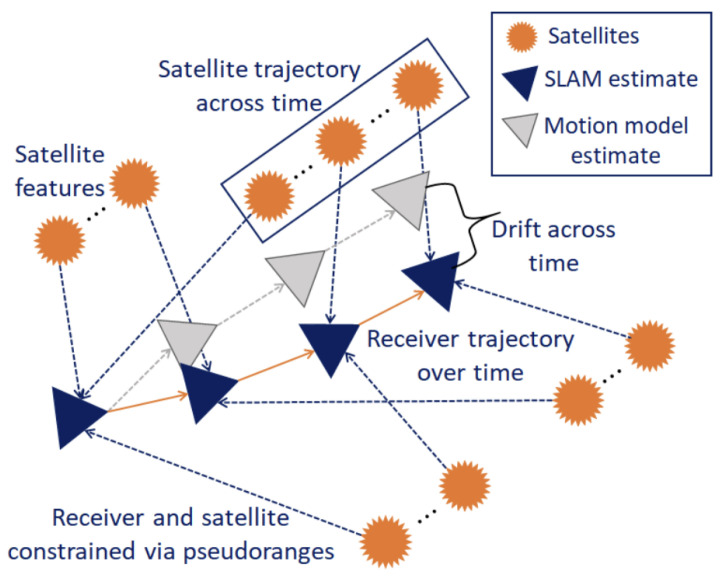
GraphSLAM-based FDE algorithm detects and excludes multiple GPS faults. Orange stars represent GPS satellite landmarks. Blue triangles show the GPS receiver trajectory estimated by the GraphSLAM-based FDE. Gray triangles depict the vehicle trajectory estimated using only its motion model. This figure was adopted from [65].

A GraphSLAM-based FDE technique is used in [65]. GPS satellites are treated as 3D landmarks by combining GPS data with the vehicle motion model in the GraphSLAM framework; see Figure 17. The algorithm creates a factor graph using pseudo-ranges, a motion model, and broadcast signals. The difference between predicted and measured pseudo-ranges is used to compute residuals, which are weighted in the graph optimization to detect and reject faulty measurements. The sample mean and covariance of these residuals form an empirical Gaussian distribution, used for FDE. New residuals must fall within a 25% region of this distribution to update the mean and covariance. The algorithm iteratively localizes the vehicle and satellites, eliminating faulty measurements, and updates the 3D map through local and global optimization steps.

**Figure 18:**
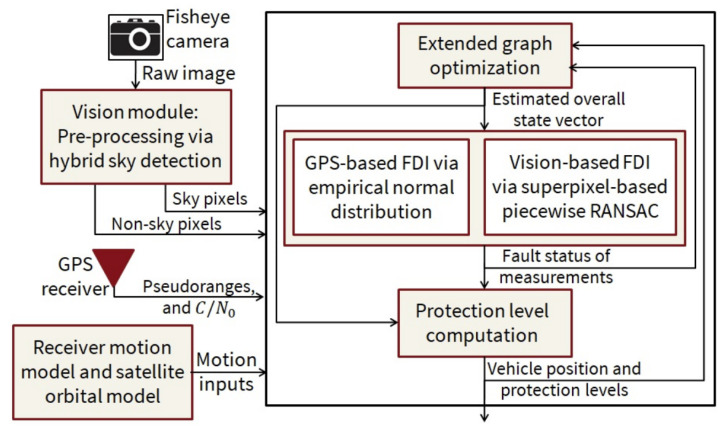
An illustration of the GraphSLAM-based integrity monitoring approach combining GPS and visual data. This figure was adopted from [47].

Building on the previous technique, the authors of [46,47] combine GPS and visual data for integrity monitoring in a GraphSLAM framework. They use GPS pseudoranges, vision pixel intensities, vehicle motion models, and satellite ephemeris to construct a factor graph. Temporal analysis of GPS residuals and spatial analysis of vision data are performed. Superpixel-based intensity segmentation [66,67], using RANSAC [68,69], labels pixels as sky or non-sky to remove vision faults. Residuals for all inputs are included in the graph optimization, with weights to detect faults and/or outliers. GPS FDE relies on temporal correlation, while vision FDE uses spatial correlation. A batch test statistic, the sum of weighted squared residuals, is computed to assess integrity. This statistic follows a Chi-squared distribution, and the protection level is calculated using the non-centrality parameter and the worst-case failure mode slope. This approach is illustrated in Figure 18.

The method in [51] enhances system integrity by estimating both the robot’s position and its reliability. According to [51], reliability is defined as the probability that the estimated pose error falls within an acceptable range. A modified CNN from [70] identifies localization failures by learning from successful and failed localizations. It converts CNN output into a probabilistic distribution using a Beta distribution [71], based on a reliability variable.

**Figure 19:**
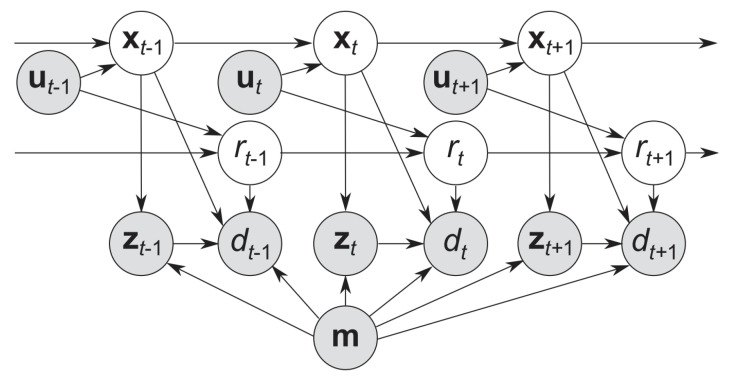
Graphical model for estimating both the robot’s current pose $x_{t}$ and the reliability, $r_{t}$, of this estimate. White nodes represent hidden variables, and gray nodes represent observable variables. The CNN uses sensor observations $z_{t}$, the map m and the pose $x_{t}$ to make a decision $d_{t}$. Reliability is treated as a hidden variable and is estimated using the CNN’s decision $d_{t}$ and the control input $u_{t}$. This figure was adopted from [51].

The conventional Dynamics Bayesian Network (DBN) model is updated with two new variables, Figure 19: the reliability variable and the CNN output. The DBN uses a Rao-Blackwellized Particle Filter (RBPF) to estimate both reliability and position. Over time, the reliability variable decays if no observations are made. To improve efficiency, ref. [52] introduces a Likelihood-Field Model (LFM) for calculating particle likelihoods. The CNN output uses a sigmoid function to indicate localization success. Positive and negative decisions are modeled with Beta and uniform distributions, respectively, with constants optimized experimentally.

Despite the LFM’s efficiency benefits, it may reduce data representation, affecting failure detection accuracy. The method also faces challenges due to the computational demands of visual data analysis. Domain-specific knowledge and computational resources are needed to determine the constants for optimal performance.

In [53], a more advanced method extends pose and reliability estimation by incorporating observed sensor measurements’ class. This approach includes three latent variables: localization state (successful or failed), measurement class (mapped or unmapped), and vehicle state. A modified LFM is used for observation sensors, and the proposed model integrates information about observed obstacles using conditional class probability [72].

In contrast to the previous methods [51,52], which employed a CNN to make decisions, this strategy uses a basic classifier based on the Mean Absolute Error (MAE) of the residual errors. The residual is the difference between the observed beams and the closest obstacle in the occupancy grid map. The final decision is computed using a threshold. This method includes both global localization using the free-space feature proposed in [73] and local pose tracking using the MCL presented in [19]. As such, it can perform relocalization due to pose tracking failure. The method in [74] is used to fuse the local and global localization approaches via importance Importance Sampling (IS).

In [44,49], a method is proposed to enhance camera-based localization in GNSS-limited areas by improving PL calculations. The approach leverages CNNs and 3D point cloud maps from LiDAR to estimate location error distributions, capturing both epistemic and aleatoric uncertainties [75,76].

**Figure 20:**
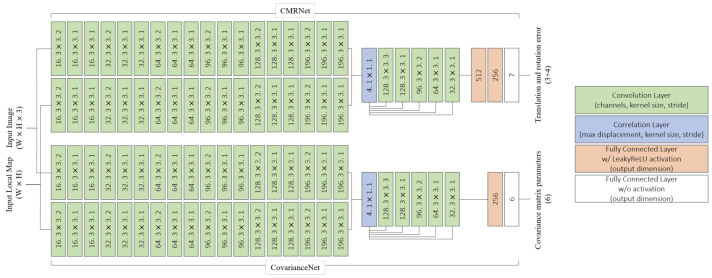
Architecture of the deep neural network for estimating error distribution using CMRNet and correlation layers. A similar architecture, CovarianceNet, is used to produce covariance matrix parameters based on the translation error output. This figure was adopted from [49].

The CNN has two components, Figure 20: one estimates position error, and the other calculates covariance matrix parameters. It uses CMRNet [77] with correlation layers [78] for error estimation and a model similar to [79] for covariance.

To handle CNN fragility, the method applies outlier weighting using robust *Z*-scores. A GMM is built from weighted error samples to represent position errors and calculate PL from the GMM’s cumulative distribution function.

However, the method’s effectiveness is limited by the need for large, high-quality training datasets, which can be labor-intensive and affect accuracy due to variability in input data and dataset quality.

#### 6.1.4. Model-Based FDE Methods: Summary and Insights

MB-FDE algorithms are promising for fault handling in localization systems but face scalability and reliability challenges. The reliance on correct sensor data, as well as the assumption of specific noise distributions, may limit their application in real-world scenarios with a wide range of environments and sensors. Practical implementation is further complicated by the computational complexity of analyzing several failure hypotheses and the combinatorial explosion in the number of measurements. Furthermore, the efficiency of these strategies is strongly reliant on the proper modeling of temporal and spatial correlations, which is not always simple or precise. Overall, while these FDE techniques are significant advances in maintaining the integrity of localization systems, more research is needed to overcome their shortcomings and increase their robustness in a variety of operating environments.

### 6.2. Coherence-Based Techniques

These techniques use the consistency of data from various sensors or localization systems to perform FDE. Fundamentally, coherence-based FDE, or CB-FDE, techniques take advantage of the idea that, in typical operational environments, several estimates of the same quantity should show coherence or agreement. These estimates can be acquired by different sensors, systems, or algorithms. Usually, the coherency check is carried out by weighing the estimates from each source and accepting the set of estimates that satisfy a threshold test. Alternatively, a test between each pair of estimate sources can be performed to check for inconsistencies.

To perform a coherence check, as shown in Figure 10, we calculate the pairwise residuals and use a coherence measure to identify the faulty measurement among $y_{1}$, $y_{2}$, and $y_{3}$. First, we define the residuals as the differences between each pair of measurements. These residuals are

(9) $$\begin{array}{cc} r_{12} & =y_{1}-y_{2},\\ r_{23} & =y_{2}-y_{3},\\ r_{13} & =y_{1}-y_{3}. \end{array}$$

These residuals represent the discrepancies between each pair of measurements. The coherence between two measurements (or residuals) can be calculated using a similarity measure, such as the cosine similarity or correlation coefficient. For simplicity, we define the coherence between $y_{i}$ and $y_{j}$ as the normalized dot product of their residuals. The coherence between $y_{i}$ and $y_{j}$ is

(10) $$\begin{array}{c} {\mathrm{Coherence}}_{ij}=\frac{r_{ij}^{T}r_{ij}}{∥r_{ij}∥∥r_{ij}∥} \end{array}$$

This coherence measure ranges from 0 to 1, where 1 indicates perfect agreement between the measurements (i.e., no fault), and 0 indicates a large discrepancy. To detect a faulty measurement, we compare the pairwise coherencies. Let the coherencies be computed as

$${\mathrm{Coherence}}_{12}=\frac{r_{12}^{T}r_{12}}{∥r_{12}∥∥r_{12}∥}$$

$${\mathrm{Coherence}}_{23}=\frac{r_{23}^{T}r_{23}}{∥r_{23}∥∥r_{23}∥}$$

$${\mathrm{Coherence}}_{13}=\frac{r_{13}^{T}r_{13}}{∥r_{13}∥∥r_{13}∥}$$

Now, we apply a threshold for coherence, γ, where measurements with coherence values below this threshold indicate a faulty measurement.

The faulty measurement is detected based on its incoherence with the others. We define a decision rule to flag the faulty measurement:

$$\mathrm{Faulty}\;\mathrm{Measurement}=\left\{\begin{array}{cc} y_{1} & \mathrm{if}\;{\mathrm{Coherence}}_{12}<\gamma \;\mathrm{and}\;{\mathrm{Coherence}}_{13}<\gamma \\ y_{2} & \mathrm{if}\;{\mathrm{Coherence}}_{12}<\gamma \;\mathrm{and}\;{\mathrm{Coherence}}_{23}<\gamma \\ y_{3} & \mathrm{if}\;{\mathrm{Coherence}}_{23}<\gamma \;\mathrm{and}\;{\mathrm{Coherence}}_{13}<\gamma \end{array}\right.$$

Here, the measurement with the lowest pairwise coherence compared to the others is flagged as the faulty one.

Once the faulty measurement is identified, it can be excluded from further processing or estimation.

$$\mathrm{Remaining}\;\mathrm{Measurements}=\left\{\begin{array}{cc} \left\{y_{2},y_{3}\right\} & \mathrm{if}\;y_{1}\;\mathrm{is}\;\mathrm{faulty}\\ \left\{y_{1},y_{3}\right\} & \mathrm{if}\;y_{2}\;\mathrm{is}\;\mathrm{faulty}\\ \left\{y_{1},y_{2}\right\} & \mathrm{if}\;y_{3}\;\mathrm{is}\;\mathrm{faulty} \end{array}\right.$$

The above example is just one case of many variations in coherence-based methods, which lie in the way coherency checks are generally performed. These variations depend on the localization algorithm or approach and the types of input utilized. The following in-depth analysis will examine a wide range of algorithms and approaches, each providing unique perspectives and techniques for CB-FDE techniques. An innovative approach for detecting localization failures is introduced by [80]. It examines the coherency between sensor readings and the map by analyzing latent variables. The model uses Markov Random Fields (MRF) [81,82] with fully connected latent variables [83] to find misalignments. These latent variables can be aligned, misaligned, or unknown, based on residual errors between measurements and the map.

The method integrates with the localization module and uses the 3D Normal Distribution Transform (3D-NDT) scan-matching technique [84,85]. It estimates the posterior distribution of latent variables from residual errors and applies a probabilistic likelihood model. The model includes a hyperparameter and selection bias. Failure probability is approximated using sample-based methods, though the precision of this approximation is not verified. The model’s multimodality, due to latent variables, may not capture all possible outcomes.

**Figure 21:**
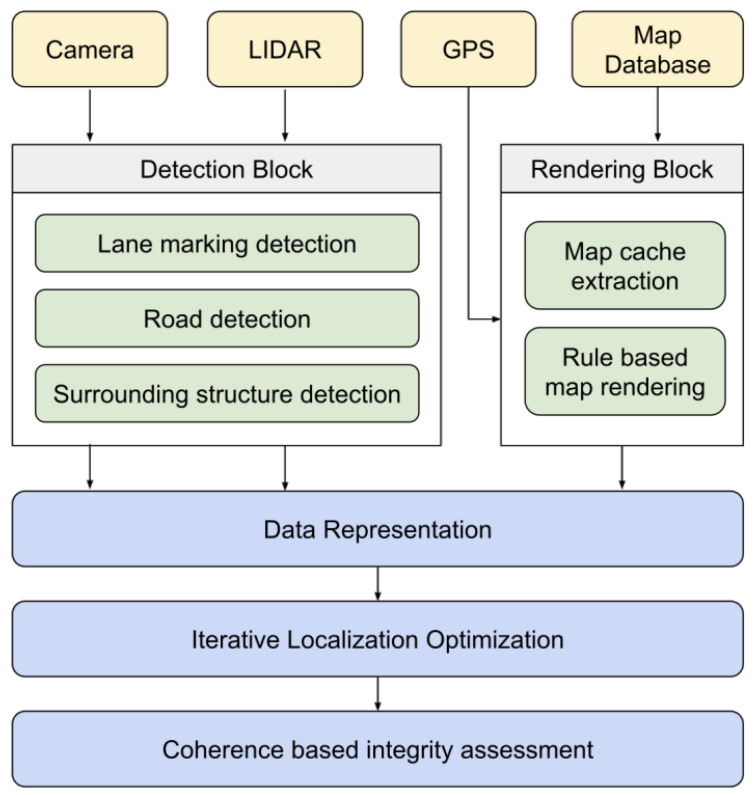
Framework for assessing integrity by ensuring consistency across multiple data sources. This figure was adopted from [37].

**Figure 22:**
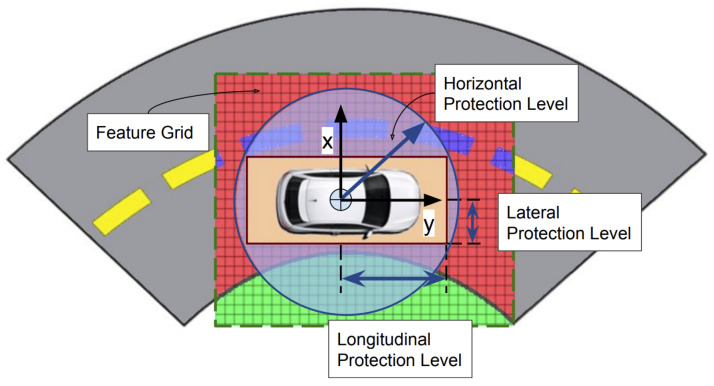
Feature grid representing the vehicle’s localization. The Feature Grid illustrates data consistency across LiDAR, camera, and map sources. It includes the detection of road surfaces (red), lane markings (blue), other surfaces (green), and unclassified points (black). PL is indicated based on the variances of particle distributions. This figure was adopted from [37].

The technique from [37] integrates data from LiDAR, cameras, and maps into a unified model, as shown in Figure 21. It maintains data consistency through redundancy and weighting. The method aligns sensor data with map data using GPS positions. A Feature Grid (FG) is used to label physical areas and assign weights based on distance. This FG model overcomes limitations of traditional geometrical models by representing different features with labels and evaluating coherence between feature grids; see Figure 22.

The particle filter from [86] is adapted for map matching, creating a uniform integrity testing framework. This approach avoids specific thresholds and does not rely on particular error noise models. The PL, including the Horizontal Protection Level (HPL), is calculated using the variances of particle distributions from sensor combinations, focusing on average standard deviation.

The technique in [87] uses multiple localization systems to detect and recover from faults. It combines an EKF with a Cumulative Sum (CUSUM) test [88] to detect faults and estimate the time when they occur. The EKF tracks outputs from various systems, comparing them to find deviations. The CUSUM test helps reduce false alarms by monitoring these deviations. When a fault is detected, the system uses stored sensor data for position estimation based on the fault time. This method does not need a specific fault model, simplifying implementation. However, it lacks the ability to exclude faults and relies on assumptions about system consistency, which may lead to false alarms or missed detections.

The approach in [89] uses EKF for sensor fusion in SLAM. It integrates multiple sensors, including MarvelMind for localization, gyroscopes, encoders, and LiDAR, with two EKFs: EKF-SLAM1 processes encoder and LiDAR data, while EKF-SLAM2 handles encoder and gyro data. Faults are detected by comparing Euclidean distances between EKF positions and MarvelMind data against a threshold. A large residual indicates a fault if it exceeds this threshold. Faults are identified by specific residual subsets, simplifying fault exclusion in sensors like gyroscopes and indoor GPS. When both the encoder and laser rangefinder fail, angular velocities are compared to find the faulty sensor. This method requires a global position estimate from Marvelmind to function correctly.

Research by [38] introduces a method for enhancing fault tolerance in multisensor data fusion systems. It uses two separate data fusion systems to ensure that only one fault occurs at a time. When a fault is detected, the system compares outputs from duplicated sensors and data fusion blocks. It measures the Euclidean distance between two EKF outputs and checks it against a preset threshold. If the distance is too high, it indicates a fault. The process involves two steps: comparing residuals and sensor outputs. Hardware faults are identified by comparing sensor outputs, while faulty localization systems are detected through residual comparison. The system recovers by using error-free localization values. However, setting detection thresholds can be expensive and context-specific.

In [90], a combined approach of model-based and hardware redundancy addresses drift-like failures in wheels and sensors. Model-based redundancy uses a mathematical model to mimic component behavior. The technique employs a bank of EKF and three gyroscopes, each assigned to specific faults, producing distinct residual signatures for fault detection. Residuals are used to identify faults in wheels, encoders, and gyroscopes. A fault is flagged if the residual exceeds three times its standard deviation for a set number of times. Simulations demonstrate the technique’s effectiveness in detecting sensor and actuator failures. However, the initial thresholds for fault detection, based on the three-sigma rule, do not adapt well to varying conditions. This can lead to false alarms or missed detections, affecting the system’s accuracy in dynamic environments.

In [91], a process model predicts the initial pose by integrating data from stereoscopic systems, LiDAR, and GPS for vehicle localization. GPS provides the absolute position, while stereoscopic systems and LiDAR estimate ego-motion. The method uses the extended Normalized Innovation Squared (NIS) test to ensure sensor coherence before data fusion. Faulty sensor observations are removed before integration. LiDAR accuracy is improved with Iterative Closest Point (ICP) and outlier rejection. Sensor coherence is checked using the extended NIS test. An Unscented Information Filter (UIF) integrates data from multiple sensors, minimizing error accumulation. Parity relations [92], calculated with Mahalanobis distance, help detect faults.

However, this method assumes only one fault occurs at a time, which may not reflect real-world scenarios. It also assumes Gaussian sensor readings, which may not be accurate in complex situations. Moreover, the Gaussian assumption may not handle noise or outliers efficiently, potentially leading to incorrect fault detection. Accurate sensor modeling and calibration are challenging, and computational costs increase with more sensors.

In [93,94], the maximum consensus algorithm localizes the vehicle. Ref. [93] uses LiDAR data, while [94] converts 3D LiDAR point clouds into 2D images. An approximate pose is aligned with a georeferenced map point cloud. The search for the vehicle’s position is limited to a predefined range, ensuring the true position is within it. Candidate positions are discretized, and a consensus set is created for each cell by counting matches between map points and sensor scan points, using a distance threshold and classical ICP cost function [95,96,97,98].

An exhaustive search finds the global optimum by identifying the transformation with the highest consensus. Despite the exponential cost with more dimensions, the search becomes constant with fixed dimensions. The algorithm covers the entire objective function distribution, and parallel processing is facilitated by simple count operations. Real-time applications benefit from discrete optimization techniques like branch-and-bound [99,100], which speed up computations.

However, ref. [93,94] have limitations due to their simplistic counting approach. It does not fully account for the importance of correspondences in vehicle pose estimation, especially in urban environments where most matches are from the ground and facades. Ref. [94] introduces a new objective function that uses normal vectors for point-to-surface matching, improving constraints, especially longitudinally. Errors are measured using the covariance matrix of position parameters, with a smaller trace (The trace of a matrix is the sum of its diagonal elements.) indicating fewer errors. Helmert’s point error, the inverse of the matrix trace, scores solution quality, guiding localization. The algorithm uses a physical beam model [19] to create a probability grid from LiDAR data, defining the PL from grid cells with a probability $p>1\times 10^{-7}$. A significant drawback is the grid-based search constraint, which limits precision to the resolution of the search space. Handling irregular point cloud distributions remains challenging, even with point-to-surface mapping.

#### Coherence-Based FDE Methods: Summary and Insights

CB-FDE techniques have a number of shortcomings. As the number of sources rises, scalability problems occur, and comparisons grow quadratically. For example, 10 sources require 45 comparisons, whereas 20 sources demand 190. This leads to increased computational complexity, making real-time problem detection difficult owing to processing delays. Additionally, these techniques rely on redundancy, which is less successful in systems with fewer sources because it requires multiple sources to provide the same value. Furthermore, as CB-FDE performs best with errors that consistently affect all sources, it is less effective with irregular error patterns.

Table 4 summarizes all MB-FDE methods and compares them using various criteria.

## 7. Robust Modeling and Optimization

FDE methods primarily focus on **qualifying** system integrity by detecting and managing faults and outliers. Qualification in this context means determining whether the system is functioning correctly by identifying when and where things go wrong. However, FDE methods often fall short in the **quantification** of integrity, which involves measuring the extent or impact of these faults. Without quantification, it is difficult to assess how errors affect the overall system performance. To address this gap, additional techniques, such as PL, are required to provide a numerical measure of system integrity.

Robust modeling and optimization techniques address both qualification and quantification. These methods do not depend on specific fault models; instead, they use general approaches to handle a wide variety of faults and noise. This allows for both a thorough assessment of whether the system is performing correctly (qualification) and a measurement of how well it is performing (quantification). By providing probabilistic interpretations of error distributions, robust methods give a more complete picture of system integrity.

In localization tasks, the common assumption that errors follow a Gaussian distribution often fails. This is not only because of linear approximations in algorithms like factor graphs and Bayesian methods but also due to other factors such as the presence of unknown outlier distributions; see Section 2.1.

These outliers can arise from various front-end [102] processes involved in building the factor graph, such as the following:

- **Image or LiDAR Scan Matching Errors** [103,104,105]: In odometry, mismatches in image sequences or LiDAR scans can introduce significant outliers.
- **Loop Closure Detection** [106]: In SLAM-based localization, incorrect identification of loop closures can distort the graph and lead to substantial errors.
- **Erroneous Map Queries** [107,108,109]: In map-based localization, errors can occur during the process of querying the map, particularly in the absence of accurate GPS data.
- **Mapping Errors** [105,110,111,112]: Outliers can also arise due to inaccuracies in the map itself, which may result from errors accumulated during the map generation process. These mapping errors can propagate through the system, leading to further mismatches during map matching and adding additional outliers.

These front-end issues, if not properly handled, can weaken both the qualification and quantification of system integrity.

Robust modeling techniques are particularly valuable in these scenarios because they can manage diverse sources of error without needing detailed models of every possible fault. Unlike traditional methods, robust algorithms dynamically adapt to uncertainties in real time, which is crucial in complex, changing environments. These algorithms effectively mitigate the impact of various uncertainties, making them essential for both qualifying and quantifying system performance.

**Figure 23:**
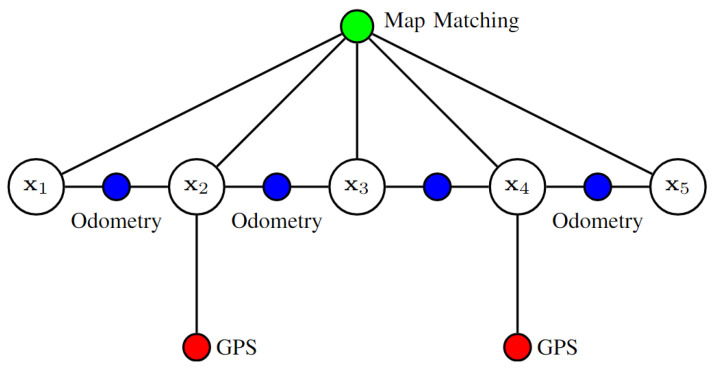
The factor graph illustrates the pose estimation problem with various constraints. The poses $x_{0}$ to $x_{5}$ are represented as circular nodes, connected by different types of factors, indicated by colored filled circles. Odometry constraints (blue) are binary factors linking successive poses, representing the motion model between consecutive states. GPS constraints (red) are unary factors applied to specific poses, reflecting time-dependent GPS measurements. The map matching constraint (green) is a multi-node factor connecting all poses, ensuring alignment with a known map. In this figure we ignore the prior over initial state.

In localization, robust modeling is often applied using a factor graph model [113,114,115], as shown in Figure 23. In this model, a graph representation is adopted, where nodes correspond to different states or poses, and edges represent constraints between them. These constraints can come from various sources, such as the following:

- **Odometry**: Using methods like ICP from image sequences or LiDAR scans, or motion models from IMU data;
- **GPS**: Providing positional constraints based on satellite data;
- **Map Matching**: Aligning sensor data with a known map;
- **Landmarks Observations**: Constraints from observing known landmarks;
- **Calibration Parameters**: Constraints related to sensor calibration.

These constraints generate residuals, and the sum of these residuals represents the total energy or loss of the graph. The goal of optimization is to minimize this loss, thereby refining the graph’s configuration to best fit the sensory information. Factor graph optimization can be performed either online, as data are received, or offline, using a batch of data.

To mathematically model this process, the optimization problem can be formalized as follows:

(11) $$\begin{array}{c} x^{*}=\min_{x}\sum_{i}{\rho (||r\left(x\right)||}_{1}) \end{array}$$

where

- $r_{i}\left(x\right)$ is the residual for the *i*-th constraint;
- $\rho_{i}\left(\cdot \right)$ is a robust kernel (e.g., Huber, Cauchy) that reduces the influence of large residuals caused by outliers.

The residuals are defined generically over manifolds as the abstract difference between measurements $z_{i}$ and predictions ${\hat{z}}_{i}\left(x\right)$. This abstract difference is expressed using the *boxminus* operator (⊟) [116] to ensure proper handling of the manifold structure. The residual for factor *i* is given by

(12) $$\begin{array}{c} r_{i}\left(x\right)={\hat{z}}_{i}\left(x\right)⊟z_{i} \end{array}$$

where

- ${\hat{z}}_{i}\left(x\right)$ is the predicted measurement based on the current estimate of the state x;
- $z_{i}$ is the observed measurement for factor *i*;
- ⊟ denotes the manifold-aware difference, which accounts for the non-Euclidean nature of the state space.

Solving Equation 12 using the iteratively reweighted least squares (IRLS) [117,118,119,120]:

(13) $$\begin{array}{c} x^{*}=\min_{x}\sum_{i}w_{i}\left|\right|r_{i}\left(x\right){\left|\right|}_{2}^{\,} \end{array}$$

where the weights $w_{i}$ are defined as

(14) $$\begin{array}{c} w_{i}=\frac{\partial ||r_{i}\left(x\right){\left|\right|}_{1}}{\partial x}\frac{\partial \rho (||r_{i}\left(x\right)|{|}_{1})}{\partial ||r_{i}\left(x\right){\left|\right|}_{1}} \end{array}$$

The optimization process alternates between solving the weighted least squares problem and updating the weights $w_{i}$ based on the residuals $r_{i}\left(x\right)$ of the current solution. This iterative process continues until convergence criteria (e.g., residual reduction or parameter update threshold) are satisfied.

By addressing both the qualification and quantification of errors, robust modeling ensures that the system not only identifies faults but also accurately measures their impact, leading to a resilient and precise localization system. This approach is particularly crucial in environments where the quality of the sensor’s data can significantly influence the overall performance.

This section reviews the primary algorithms and techniques used to create robust localization algorithms.

### 7.1. Analysis of Robust Modeling and Optimization Techniques

Current localization methods often use least squares optimization but face challenges when dealing with outliers like data association errors and false positive loop closures.

**Figure 24:**
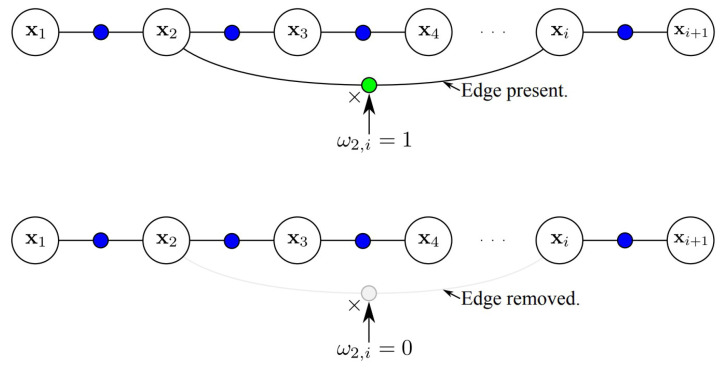
A binary weight $\omega_{2,j}\in \left\{0,1\right\}$ is assigned to each loop closure constraint. When $\omega_{2,j}=1$, the constraint remains active (top). When $\omega_{2,j}=0$, the constraint is either disabled or removed (bottom). If these weights are **treated as variables in the optimization process**, the constraints can be adjusted or excluded during optimization. This figure was adopted from [124].

To address these issues, ref. [121] introduced a solution that improves the back-end optimization process. Instead of keeping the factor graph structure or topology fixed, this method allows the graph topology to change dynamically during optimization. This flexibility helps the system detect and reject outliers in real time, making SLAM more robust. The method uses *switch variables*, see Figure 24, for each *potential* outlier constraint or edge. These variables help the system decide which constraints to include or exclude based on their accuracy. Essentially, switch constraints (SC) act as adjustable weights for each factor in the factor graph. These weights are optimized along the map for SLAM [121] and the pose for GNSS-based localization [122,123].

This approach is similar to FDE because it automatically identifies and removes erroneous data associations or pseudo measurements, ensuring the system uses only reliable data [121,122,123]. However, this approach introduced extra switch variables for each potential outlier, which increased both the computational cost and complexity of each iteration. As a result, the system could become less efficient and slower to converge.

In contrast, Dynamic Covariance Scaling (DCS) [125,126,127,128] offers a more efficient method for managing outliers in SLAM without adding extra computational load. DCS adjusts the covariance of constraints based on their error terms, changing the information matrix without needing additional variables. This makes the optimization process more efficient and speeds up convergence. The scaling function in DCS is determined analytically and is related to weight functions in *M*-estimation [129], which reduces the number of parameters to estimate compared to the SC approach, since DCS does not require iterative optimization of the scaling function.

The earlier methods using SC and DCS had challenges with tuning the scaling function based on error and also required manual adjustment of parameters [130]. The method in [131] addresses this issue with self-tuning M-estimators. This approach directly adjusts the parameters of M-estimator cost functions, which simplifies the tuning process.

The self-tuning M-estimators method connects M-estimators with elliptical probability distributions, as introduced in [131]. This means that M-estimators can be chosen based on the assumption that errors follow an elliptical distribution. The algorithm then automatically adjusts the parameters of the M-estimators during optimization, selecting the best one based on the data’s likelihood.

**Figure 25:**
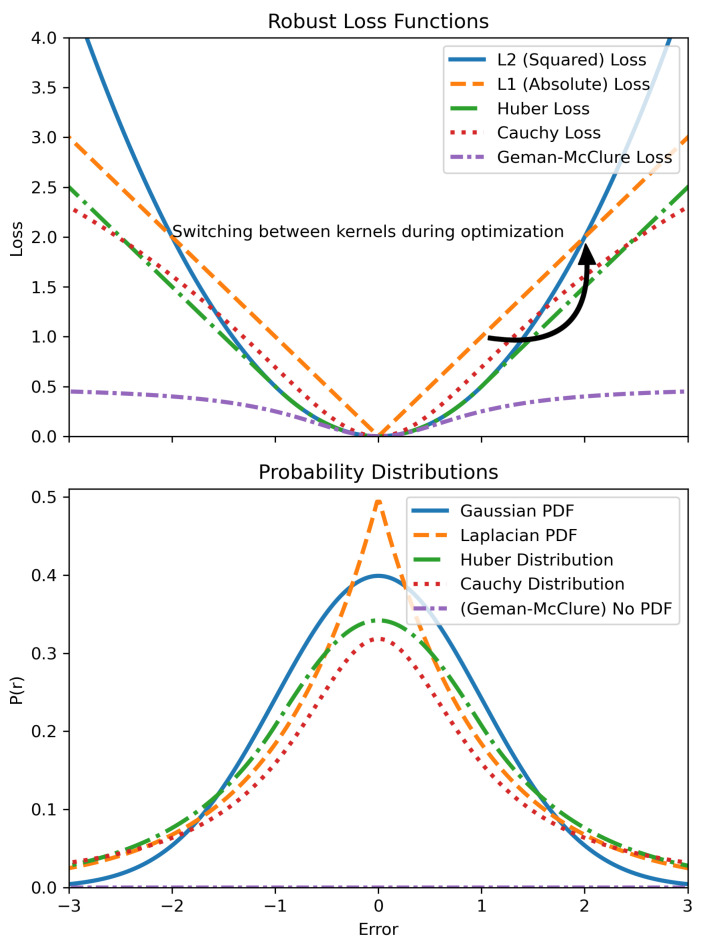
The top plot displays the robust loss functions: L2 (Squared) loss, L1 (Absolute) loss, Huber loss, Cauchy loss, and Geman–McClure loss, each depicted with distinct line styles. The bottom plot shows the associated probability distributions, including Gaussian PDF for L2 loss, Laplacian PDF for L1 loss, and the specific distributions for the other kernels. Notably, Geman–McClure loss has no associated probability distribution, represented by a horizontal zero line.

A broader approach to robust cost functions is introduced in [132]. This method improves algorithm performance for tasks like clustering and registration by treating *robustness as a continuous parameter*. The robust loss function in this framework can handle a wide range of probability distributions, including normal and Cauchy distributions, by using the negative log of a univariate density. **By incorporating robustness as a latent variable in a probabilistic framework, this approach automatically determines the appropriate level of robustness during optimization, which eliminates the need for manual tuning and provides a more flexible solution**. Building on this, ref. [133] presents a method that dynamically adjusts robust kernels based on the residual distribution during optimization. This dynamic tuning improves performance compared to static kernels and previous methods. The key difference from [132] is that the new method covers a wider range of probability distributions by extending the robust parameter’s range. The shape of the robust kernel is controlled by a hyperparameter that adjusts in real time, enhancing both performance and robustness. Figure 25 represents various robust kernels and the switching between them during optimization. *The switching between these kernels during optimization occurs due to the fact that robustness is modeled by an optimizable latent variable*.

In contrast, ref. [134] introduces a probabilistic approach to improve convergence. This method fits the error distribution of a sensor fusion problem using a multimodal GMM. In real-time applications like GNSS localization, the adaptive mixing method adjusts to the actual error distribution and reduces reliance on prior knowledge. This approach effectively handles non-Gaussian measurements by accurately accounting for their true distribution during estimation. In sensor fusion, where asymmetric or multimodal distributions are common, this method provides a probabilistically accurate solution. It uses a factor graph-based sensor fusion approach and optimizes the GMM adaptation with the Expectation Maximization (EM) algorithm [135,136].

In [44,137], a robust multisensor state estimation method combines a Particle Filter (PF) with a robust Extended Kalman Filter (R-EKF) using RBPF. It replaces the Gaussian likelihood with robust cost functions like Huber or Tukey biweight loss [129] to better handle non-Gaussian errors and outliers unlike standard EKF methods [138,139]. The approach uses PF for linearization points and R-EKF for state estimation, integrating estimates across points with resampling [140]. Position error bounds are estimated using a GMM and Monte Carlo integration [19], addressing orientation uncertainty and providing robust probabilistic bounds. Limitations include sensitivity to initial parameters, potential convergence to local optima, and added complexity in tuning and balancing loss terms, which can introduce biases or errors.

### 7.2. Robust Modeling and Optimization: Summary and Insights

In conclusion, this section underscores the importance of both FDE techniques and robust modeling approaches for enhancing the integrity of localization systems. FDE qualifies system integrity by managing faults and deviations but doesn’t measure their impact on performance. Robust modeling qualifies and quantifies integrity by handling errors and providing probabilistic error bounds. Specifically, robust modeling offers two key features: it associates a probabilistic distribution over the error and dynamically accounts for variations in system uncertainty as described in [132,133]. Thus, to fully address the integrity of the localization system, integrating FDE or robust modeling with PL is essential.

Lastly, while PL quantifies integrity, the evaluation of integrity varies across applications. This typically involves computing the Integrity Risk (IR) and comparing it with the Total Integrity Risk (TIR) to determine the likelihood of the true position exceeding the provided PL, as described in Section 5.

Table 5 summarizes all the robust methods and compares them using various criteria.

## 8. Conclusions

In conclusion, this survey paper presents several significant contributions to the field of integrity methods in localization systems. It identifies a crucial gap in the research on integrity methods for non-GNSS-based systems, highlighting the need for more efforts in this area.

While 73.3% of surveyed literature focuses on GNSS-based systems, only 26.7% covers non-GNSSs that use various sensors and approaches, such as cameras, LiDAR, fusion, optimization, or SLAM. Furthermore, among these, only a small fraction specifically explores protection level calculations.

This paper introduces a unified definition of integrity that encompasses both qualitative and quantitative aspects, cf. Section 4. The new definition integrates robustness, outlier management, and deviation measures, providing a holistic evaluation of localization systems. The proposed framework improves upon existing definitions by offering a comprehensive view that includes the system’s alignment with reality and detailed error handling.

The survey reviews and refines the definitions of PL, cf. Section 5. It points out that current definitions do not account for all uncertainties and limitations from system components and algorithms. The new definition of PL provided here addresses these gaps by requiring a real-time estimate of PL. This definition facilitates effective adjustment to changing environments and sensor conditions, ensuring real-time system integrity assessment.

The survey provides a detailed review of FDE methods. The FDE techniques are categorized into model-based and coherence-based approaches, examining their applications, effectiveness, and limitations. Model-based FDE methods are further divided into post-estimation, pre-estimation, and integrated-processing categories. While these methods are promising for fault handling, they face challenges related to scalability, reliability, computational complexity, model selection, and accurate modeling. Coherence-based FDE techniques also encounter issues with scalability and effectiveness, particularly with irregular error patterns.

Moreover, the paper introduces robust modeling and optimization as essential methods for integrity. Unlike traditional FDE methods that focus primarily on qualification, robust modeling addresses both qualification and quantification. It provides probabilistic error bounds and adapts to variations in system performance, offering a more comprehensive view of integrity. This approach allows for a thorough assessment of system performance and measurement of how well it is functioning.

Finally, the survey includes comparative tables that summarize and evaluate various integrity methods, highlighting their strengths and limitations. This comparative analysis provides a clearer understanding of how different methods can be applied across various localization systems.

Overall, this paper offers a valuable reference for researchers and practitioners, presenting a detailed review of integrity methods, new definitions, and a comprehensive classification framework. It sets a foundation for future research and development, aiming to enhance the safety and efficiency of localization technologies by addressing key gaps and offering a more complete understanding of integrity and protection levels.

## Figures and Tables

**Table 1:** Summary of model-based fault detection and exclusion methods ^1^.

Reference	Algorithm	Fault Detection	Fault Exclusion	PL	Evaluation	Data	Sensor
[25,26]	EIF	Residual calculation using Mahalanobis distance	Compare residual with Chi-square distribution	Adjust covariance for estimated error using Student *t*-distribution	Calculate IR and compare with TIR	Data gathered for city Rambouillet	Odometry, GNSS, camera, HD map
[27]	*t*-EIF	Residual calculation using Kullback–Leibler Divergence	Compare residual with Chi-square and F-distributions	Compute PL with minimum degree of freedom	Calculate IR and compare with TIR	Data gathered for town of Compiegne	Odometry, GPS, camera, HD map
[45]	Particle filter	Use selection vector to vote for faulty measurement	Exclude faulty measurements	Use GMM to calculate error probability	Compare PE with PL	Simulation and Chemnitz data	GNSS, odometry
[41,42]	EKF	Compute state residual error and compare with Chi-square distribution	Weight sensors based on residual error	Use Chi-square distribution for misdetection probability	N/A	Data acquired in urban context	Wheel speed sensors, yaw rate gyroscope, GPS
[39]	EKF	Feature-based approach	Dynamic thresholding	Use EKF error bound for error covariance	Miss detection and false alarm rate	Data acquired in urban canyons in Beijing	GNSS, INS, LiDAR
[46,47]	GraphSLAM	Test statistic computation, RANSAC	Batch test statistic computation	Perform worst-case failure slope analysis	Compare PE with PL	Data collected in alleyway of Stanford and semi-urban area of Champaign, Illinois	GPS, fish eye camera
[48]	UKF	Hotelling’s $T^{2}$ test, Student *t*-distribution	Compare with a threshold	N/A	Compare with the standard UKF, adaptive UKF, adaptive UKF with the proposed FDE, and *t*-student adaptive UKF with the proposed FDE	Simulation and Highway experimental scenario	GNSS, IMU, velocity wheel sensor, steer angle, and position and azimuth using a SLAM
[34]	EKF	Parity space test	Compare residual with Chi-square distribution	Perform worst-case failure slope analysis	Compare slope of position error with respect to test statistics of parity space test	Simulation data	INS, camera
[49]	CMRNet	Outlier weighting	N/A	Cumulative distribution function of a GMM	Bound gap, false alarm rate and failure rate	KITTI visual odometry dataset [50]	Camera

^1^ All vehicles are ground vehicles.

**Table 2:** Summary of model-based fault detection and exclusion methods ^1^.

Reference	Algorithm	Fault Detection	Fault Exclusion	PL	Evaluation	Data	Sensor
[51]	RBPF + CNN	Detection of localization failure using CNN	N/A	N/A	Compare the results with AMCL ^2^	Simulation data and real indoor experiments	LiDAR
[52]	RBPF + CNN + LFM	Detection of localization failure using CNN	N/A	N/A	Compare the results with AMCL	Experimental environment and simulation environment	LiDAR
[53]	Free-space feature + MCL + IS	Detection of localization failure using MAE	N/A	N/A	Compare the results with AMCL	Simulation environment, robotics 2D-Laser datasets ^3^	LiDAR
[54]	VO-EKF	FDE using hierarchical clustering	Distance threshold between the pseudo range and the class center	N/A	Compare the result to the same system but without the FDE step	Field test Nanjing, Jiangsu, China, with the raw GNSS measurement	GPS, IMU, and binocular depth stereo camera
[55]	B-CIIF	Decision tree	Random forest	N/A	Accuracy of the decision tree and random forest	Experimental environment	Wheel encoders, IMU, LiDAR, and Marvelmind system

^1^ All vehicles are ground vehicles. ^2^ Augmented MCL [19,56,57]. ^3^ https://www.ipb.uni-bonn.de/datasets/ (accessed on: 1 December 2024)

**Table 3:** Summary of model-based fault detection and exclusion methods ^1^.

Reference	Algorithm	Fault Detection	Fault Exclusion	PL	Evaluation	Data	Sensor
[35,36]	ORB-SLAM2	Parity space test	Compare residual with Chi-square distribution	Weighted covariance for sensor noise	Compare PL with 3σ	EuRoC dataset	Camera
[58]	B-CIIF	MLP	MLP	N/A	Accuracy of the MLPs	Experimental environment	Wheel encoders, IMU, LiDAR and Marvelmind system
[59]	EIF	GKLD measure between prediction and update distribution	Use EIF bank for fault exclusion	N/A	Compare FDE with ground truth trajectory	Indoor environment	Wheel encoders, gyroscope, Kinect, LiDAR
[60]	EIF	Jensen Shannon divergence compared to Youden index of ROC curve	Signature matrix-based exclusion	Not specified	Data acquired by three Turtlebot3	Experimental environment	Wheel encoders, IMU, LiDAR, Marvelmind system

^1^ All vehicles are multi-ground vehicles except for [35,36], which are micro aerial vehicles.

**Table 4:** Summary of coherence-based fault detection and exclusion methods ^1^.

Reference	Algorithm	Coherency Check	PL	Evaluation	Data	Sensor
[37]	Particle filter-based map-matching	FG cell’s weight is used to weight each source and a threshold is applied to detect the incoherent source	HPL determined by the variances of particle distributions from each sensor combination used in the localization algorithm	Compare the HPL calculated by this method with historical values in [101]	KITTI with different scenarios	Cameras, LiDAR, GPS
[80]	3D-NDT	MRF that exploits the full correlation between the sensor measurement	N/A	Root Mean Square error	SemanticKITTI dataset, and data acquired on Japanese public roads	LiDAR
[87]	EKF	Cumulative Sum test	N/A	Observing when a faulty localization system is detected	Data acquired by the vehicle	Odometry and LiDAR
[89]	EKF-SLAM	Euclidean distances between positions from the EKFs and MarvelMind are measured and compared to a predetermined threshold	N/A	Compare the obtained pose with the ground truth trajectory	Acquired data through an experimental environment	EKF1 (encoder and LiDAR), EKF2 (encoder and gyroscope), Marvelmind
[38]	EKF	Euclidean distance between the two EKF systems output is calculated and compared with a threshold	N/A	False positive rate, no detection rate, detected errors rate, etc.	Data acquired in the city of Compiègne, France; also, a simulation environment by using real data processed offline	INS, GPS, wheel sensors, gyrometer, and steering angle sensor
[90]	EKF	8 residuals are generated and the one that exceeds 3σ for a specific number of time steps is excluded	N/A	False alarm	Simulation models	Gyroscopes and wheel encoders
[91]	UIF	Extended NIS test where the sensor of increased residual is excluded	N/A	Compare with the ground truth trajectory	Real data acquired by an experimental vehicle	GPS, stereoscopic system, LiDAR
[93,94]	Maximum consensus	A consensus set for each pose candidate	Subset of grid cells that together account for a probability $p>1-10^{-7}$	N/A	Measurement data were recorded in an inner-city area with a dense building structure	LiDAR

^1^ All Vehicles are Ground Vehicle.

**Table 5:** Summary of Robust Algorithms ^1^.

Reference	Algorithm	PL	Evaluation	Data	Sensor
[137]	RBPF R−EKF	Compute the maximum quantile for a specific TIR	Compare with base lines such as EKF with GNSS measurements only, EKF with MD-VO ^2^ only and EKF with GNSS measurements and MD-VO	Hong Kong UrbanNav dataset [141]	GNSS, LiDAR, camera, and IMU
[121]	Factor Graph+ SC	N/A	Compare with the ground truth	Synthetic, like Manhattan, and real-world datasets, e.g., Intel	Odometry and Camera
[122]	Factor Graph + SC	N/A	Compare with the ground truth	real dataset	Odometry and GNSS receiver
[125]	Factor Graph+ DCS	N/A	Compare with the results of SC [122]	Synthetic, like Manhattan, and real-world datasets, e.g., Intel	Odometry and Camera
[131]	Factor Graph + Self tuning M-Estimator	N/A	Compare the normalized squared error for different M-estimators	real data	Four monocular fish-eye cameras
[133]	ICP + Bundle Adjustment	N/A	Compare with static kernels, with [132] as well as SuMa ^3^ [142]	KITTI for ICP and CARLA simulator [143] for bundle adjustment	LiDAR for ICP, and camera for bundle adjustment
[134]	Factor Graph + EM	N/A	Chemnitz City and smartLoc [144] dataset	N/A	GNSS receiver and odometry

^1^ All Vehicles are Ground Vehicle. ^2^ Monocular-Depth Visual Odometry (MD-VO) ^3^ A dense mapping approach called Surfel-based Mapping (SuMa).

## Data Availability

Not applicable.
